# Polymer-based heterojunction photocatalysts for environmental remediation and solar fuels: a review on interface engineering, stability, and sustainable design

**DOI:** 10.1039/d6ra07665c

**Published:** 2026-10-26

**Authors:** Abdul Majid, Muhammad Tayyab Raza, Anzal Noureen, Harram Najum, Emaan Shahid

**Affiliations:** a Department of Physics, Hafiz Hayat Campus, University of Gujrat Gujrat Pakistan abdulmajid40@yahoo.com; b Department of Physics, Gachon University 1342 Seongnam-daero, Sujeong-gu Seongnam-si Gyeonggi-do South Korea ali1214@gachon.ac.kr

## Abstract

Polymer-based heterojunction photocatalysis is a rapidly emerging paradigm in sustainable chemical engineering. It has appeared as a potential platform for tackling the concurrent global problems of environmental remediation and solar fuel production. These composite architectures overcome the inherent limitations of conventional photocatalysts, such as high carrier recombination rates, limited visible-light absorption, and poor catalyst reusability. This review aims to critically and comprehensively cover the basic photophysical and interfacial mechanisms that underpin polymer-based systems. We systematically monitor the entire photocatalytic cycle from the initial chromophore excitation and exciton generation on the polymer to state-of-the-art charge separation at donor–acceptor networks and Z-scheme heterojunction interfaces. Advanced synthesis and surface engineering techniques, such as chemical polymerization, nanostructuring, and green scalable synthesis pathways, are of particular interest, along with the strategic tuning of the Highest Occupied Molecular Orbital (HOMO) and Lowest Unoccupied Molecular Orbital (LUMO) boundary potentials in π-conjugated and metal-free frameworks to maximize the solar spectral utilization. In addition, critical chemical engineering applications involving the degradation of organic pollutants in the presence of sunlight, purification of ambient air, evolution of H_2_ with the help of solar energy, and CO_2_ reduction with the help of sunlight are presented in terms of structure–property–performance relationships of these emerging architectures. Finally, we summarize the current technical challenges for large-scale implementation and provide strategic analysis on the limitations of mass transport, operational stability in long periods, and scalable engineering design rules for future applications in environmental and energy fields at industrial scale.

## Abstract 2

Polymer Z-scheme heterojunctions utilize tailored interfaces for spatial charge separation, enabling visible-light-driven environmental remediation (pollutant degradation, air purification) and solar fuel synthesis (H_2_ evolution, CO_2_ reduction). Image created using Google Gemini.

## Graphical abstract

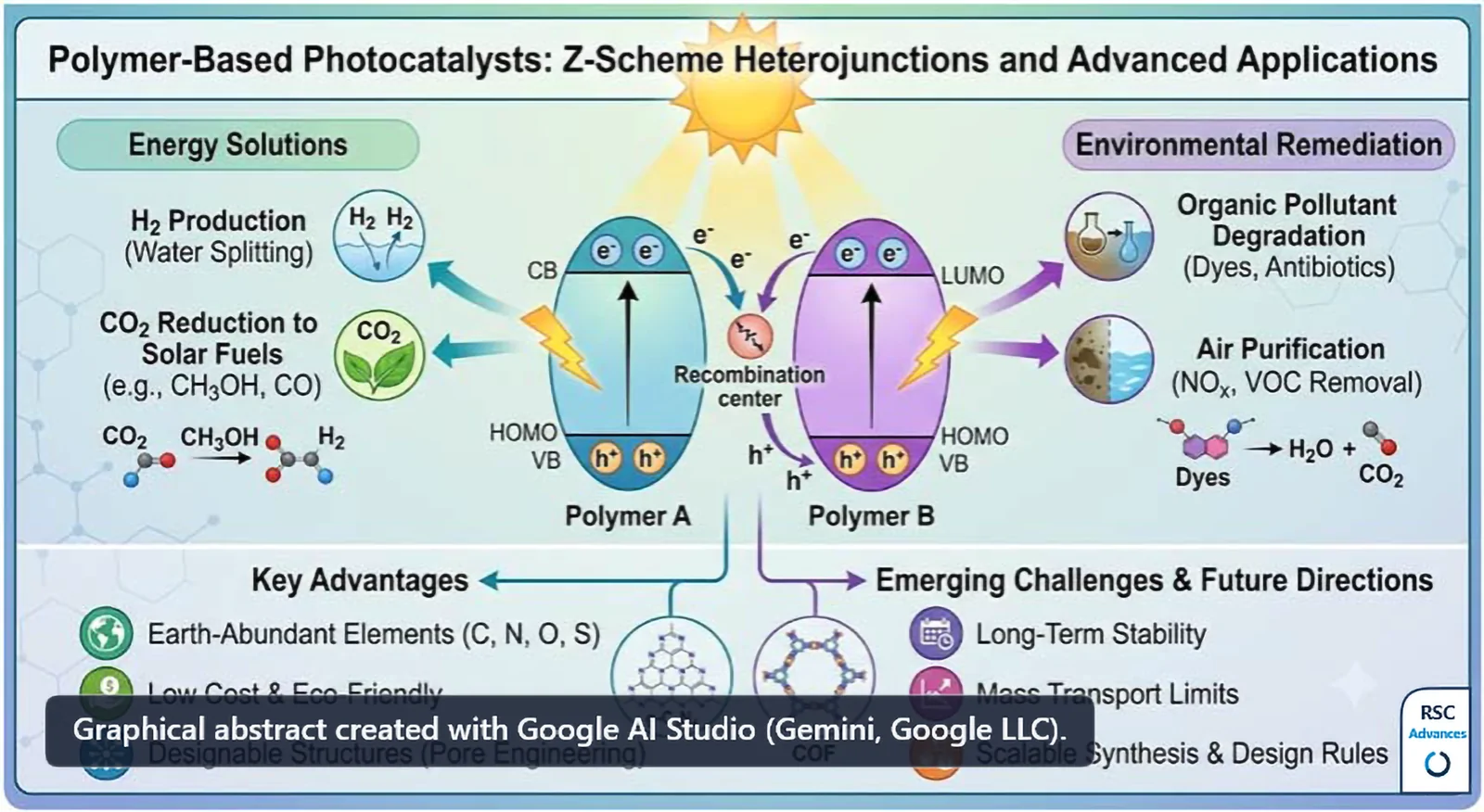

## 1 Introduction

In recent years, semiconductor-based heterojunction photocatalysis has become a key technology for addressing environmental depletion and energy shortages. This sustainable process is based on solar irradiance, which can be used for advanced oxidation processes (AOPs) and fuel-generation pathways, generating green chemical energy by using the energy of photons with minimum environmental impact.^1,2^ The focus of this research area has been on metal oxides, such as titanium dioxide (TiO_2_), and metal chalcogenides, such as cadmium sulfide (CdS), which are characterized by their wide bandgap and narrow bandgap, respectively, for decades. However, the classical catalyst materials such as TiO_2_ have significantly limited industrial applications because of their large optical band gap (∼3.2 eV), which absorbs only UV, slow charge carrier separation rates, high exciton recombination and difficult catalyst recovery after liquid-phase processing.^3,4^ To overcome these thermodynamic and kinetic restrictions, this review proposes a novel design paradigm for photocatalytic architectures using polymers. In these architectures, active semiconductor domains are embedded in tailored polymeric matrices. These matrices range from natural biopolymers (cellulose, chitosan) to conducting polymer networks such as polystyrene (PANI) and hydrogels.

One approach to provide the photocatalyst in the polymer matrix is by physically mixing the catalyst with the polymer, *in situ* synthesis, or by surface deposition. The photocatalyst creates electron–hole pairs upon light absorption and these pairs facilitate redox reactions for the degradation of pollutants, water splitting, or organic synthesis. In 2009, a big step forward was made with graphitic carbon nitride (g-C_3_N_4_), a metal-free polymer that can catalyze water splitting under visible light without the use of metal co-catalysts.^1^ This finding has led to research in lightweight metal-free porous crystals, such as Conjugated Microporous Polymers (CMPs) and Covalent Organic Frameworks (COFs).^2^ More recent developments involve donor–π–acceptor (D–π–A) polymers for better charge separation and polymer–inorganic heterostructures for higher charge mobility and stability.^3^ The usage of a polymer base offers several benefits compared to using the photocatalyst in powder form, including enhanced recoverability and reusability, improved dispersion and stability, and provision of tunable properties. Thus, polymer-based photocatalysis is a strategic way to develop the practical use and the efficiency of photocatalytic processes, exploiting the structural and functional features of polymeric materials. Several reviews already address polymer-based photocatalysts. Some focus on a single materials class, such as g-C_3_N_4_, COFs, or CMPs^4^ while the others are concerned with a single application like water splitting, CO_2_ reduction or the degradation of pollutants.^5–7^ Still others focus on synthesis methods or mechanistic aspects in isolation.^6^ To the authors' knowledge, none of these reviews consistently applies the same set of criteria for structure, charge transport, stability, and sustainability across all of the major polymer classes. This has left the performance comparison of different polymer classes inconsistent; for example, a conducting polymer heterojunction *versus* a covalent organic framework heterojunction.

This review fills this gap in five different ways. First, it compares the charge dynamics of conducting polymers, conjugated polymers, metal-free polymers and emerging classes using the same set of parameters. Second, it distinguishes interface-engineering claims that are experimentally demonstrated from those that are only indirectly inferred. Third, it explores the relationship between interface architecture and charge separation, migration and surface reaction rates. Fourth, it carries out an evaluation of stability beyond photo-corrosion alone. This includes leaching, fouling, synthesis energy, precursor impact, and techno-economic viability. Fifth, it outlines sustainable design beyond the metal-free aspect and also includes synthesis energy, precursor impact, scale-up potential and long-term stability. These five contributions connect structure, interface, charge dynamics, performance, stability and sustainability together and present a unified structure for next-generation polymer photocatalysts.

The agreements, contradictions and gaps in the literature are highlighted throughout the review and are used to prioritize future work. Fig. 1 represents a roadmap of the shift from conventional inorganic semiconductor powders to tailored polymer-based photocatalytic matrices, highlighting the core limitations of traditional photocatalysts.

**Fig. 1:**
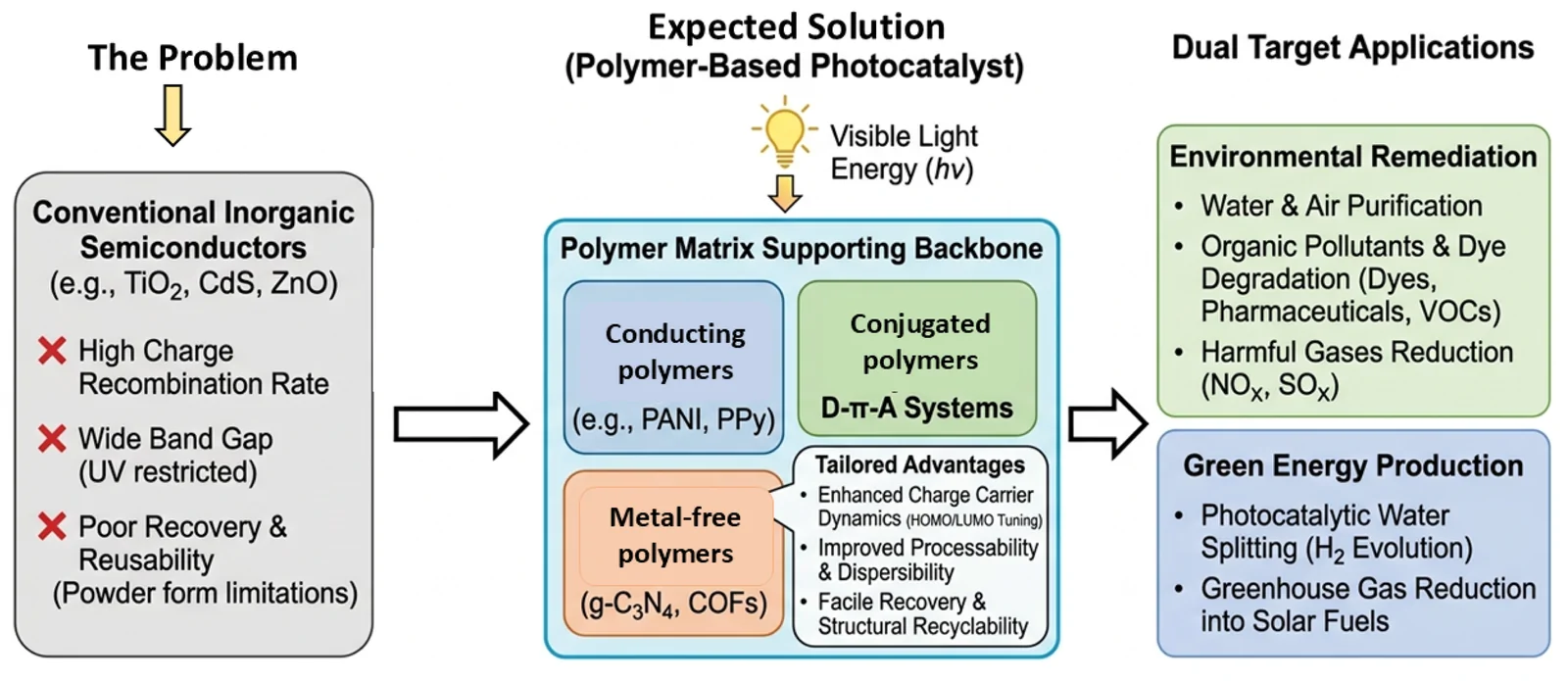
Schematic illustration of the shift from conventional inorganic semiconductor powders to tailored polymer-based photocatalytic matrices, highlighting the core limitations of traditional photocatalysts, the key advantages of polymer-based systems, and their dual-target applications in environmental remediation and green energy production.

The above-mentioned features of polymer-based photocatalysts make them promising candidates for the future. This review considers the structure–function relationship of polymer-based heterojunctions, spanning structure–interface–charge dynamics–function–stability–sustainability. A comprehensive and coherent structure of key polymer families is presented, highlighting methodological limitations, consensus, conflicts, and areas for further investigation. Thus, to maintain the focus, the review is mainly centered on polymer-based heterojunction photocatalysts. The discussions on individual polymer classes (conducting, conjugated, and metal-free polymers), polymer synthesis, and related technologies (such as photocatalytic fuel cells) are focused. The information about heterojunction design, charge-transfer behaviour, or comparative performance, and relevant discussion as stand-alone topics is not intended in the scope of this work.

## 2 Core foundations of polymer-based photocatalysts

The basic steps in polymer-based photocatalysis include the absorption of light, generation of electron–hole (e^−^/h^+^) pairs, separation and migration of the charges, and surface redox reactions. When irradiated with photons of energy higher than the band gap, electrons are excited from the valence band to the conduction band, resulting in the production of electron–hole pairs in the semiconductor. Charge recombination strongly limits the photocatalytic efficiency; the photogenerated electrons and holes may recombine before they can be involved in the surface reactions, which dissipates the absorbed energy as heat/light.^8^ The excited states are different in nature and energy depending on the molecular structure, the length of the conjugation, and the degree of ordering of the polymer. The π-electron system is extended over the polymer backbone in conjugated polymers, making light absorption and charge transport along the polymer highly efficient. Their band gap can be tuned by changing the structure of the monomers, incorporation of donor–acceptor structures or by increasing the length of the conjugated structures to cover a broad range of the UV/near IR spectrum.^9^

To be photocatalytic, the excitons have to split into free charge carriers. There are several mechanisms in which this dissociation is facilitated: presence of charge polarization in donor–acceptor (D–A) systems, which will generate an internal electric field, driving the charge dissociation due to the higher difference in electronegativity between the donor and the acceptor units; presence of heteroatoms and co-catalyst nanoparticles that will create charge dissociation centers and trap sites; and presence of polymer–inorganic interfaces that will create the pathway for directional charge transfer and reduce the possibility of charge recombination. The efficiency of exciton dissociation can be determined by the exciton binding energy, and the smaller the exciton binding energy, the more efficient the charge separation. The charges thus migrated will reach the surface active sites, where they will undergo a redox reaction.^10^

These photogenerated electrons (reducing agent) and holes (oxidizing agent) move to the surface of the catalyst, and then the redox reactions take place. Electrons transform molecular oxygen (O_2_) into superoxide radicals (˙O_2_^−^), and holes convert water or hydroxide ions (OH) into hydroxyl radicals (˙OH). These are the reactive oxygen species (ROS), which mainly break down pollutants. The reduction potential in the LUMO should be sufficiently negative to enable the reduction of O_2_ to ˙O_2_^−^ (*E*° = −0.33 V *vs.* Normal Hydrogen Electrode (NHE)) and the oxidation potential in the HOMO should be sufficiently positive to allow the oxidation of H_2_O to ˙OH (*E*° = +2.80 V *vs.* NHE) or the oxidation of OH– to ˙OH (*E*° = +2.38 V *vs.* NHE). Thus, the position of HOMO and LUMO energy levels in relation to these redox potentials will be a key factor in photocatalytic activity.

It is an important principle in organic photocatalysis that the design of D–A systems is crucial. The electronic band architecture of polymeric photocatalysts is strongly modulated by the dynamics of the interfacial and intramolecular charge transfer (ICT). The donor and acceptor subunits are arranged in such a way that the difference in their electronegativities causes a structural hybridization of the molecular orbital stabilizing the HOMO and the molecular orbital destabilizing the LUMO at the same time. This synergistic decrease in the intrinsic optical bandgap significantly reduces the threshold excitation energy, and the material's photoresponse is effectively extended to the visible and near-infrared (NIR) spectral range. At the same time, the localized polarization of charge at the interface generates a strong internal electric field, which inhibits radiative exciton annihilation and facilitates the separation of the carriers in space. Once the migration to the surface occurs, the redox reactions are achieved at much localized high-activity sites, most often occupied by single heteroatoms or immobilized co-catalyst nanoparticles (*e.g.* transition metal oxides, metal sulfides). Such co-catalysts are intimately coupled and form low-resistance heterointerfaces with the host polymer matrix, resulting in an interface energy alignment that is a very strong thermodynamic driving force for continuous charge injection and greatly accelerating the surface reaction kinetics.^15^ Several intrinsic and extrinsic factors are responsible for the efficiency of charge separation and migration. Intrinsic factors include the level of crystallinity, which guides the level of charge mobility by ordered π–π stacking interactions; molecular weight and chain length, which will guide the level of conjugation or charge transport pathways; and defects and traps, which can either enhance or inhibit charge migration. The type of electrolyte or solvent can have a more or less significant effect on the conformation of the polymer chains and the interchain interactions; the temperature can affect the mobility of the charge and the recombination kinetics; the presence of electron donors or acceptors can affect the efficiency of charge separation.^11–13^ The relationship between these factors and the performance of photocatalysts must be understood and optimized to design high-performance polymer-based photocatalysts. In polymer-based photocatalysis, all the charge carrier dynamics and surface reaction processes occur in polymers, which is summarized in Fig. 2, showing the key mechanistic steps of polymer-based photocatalysis.

**Fig. 2:**
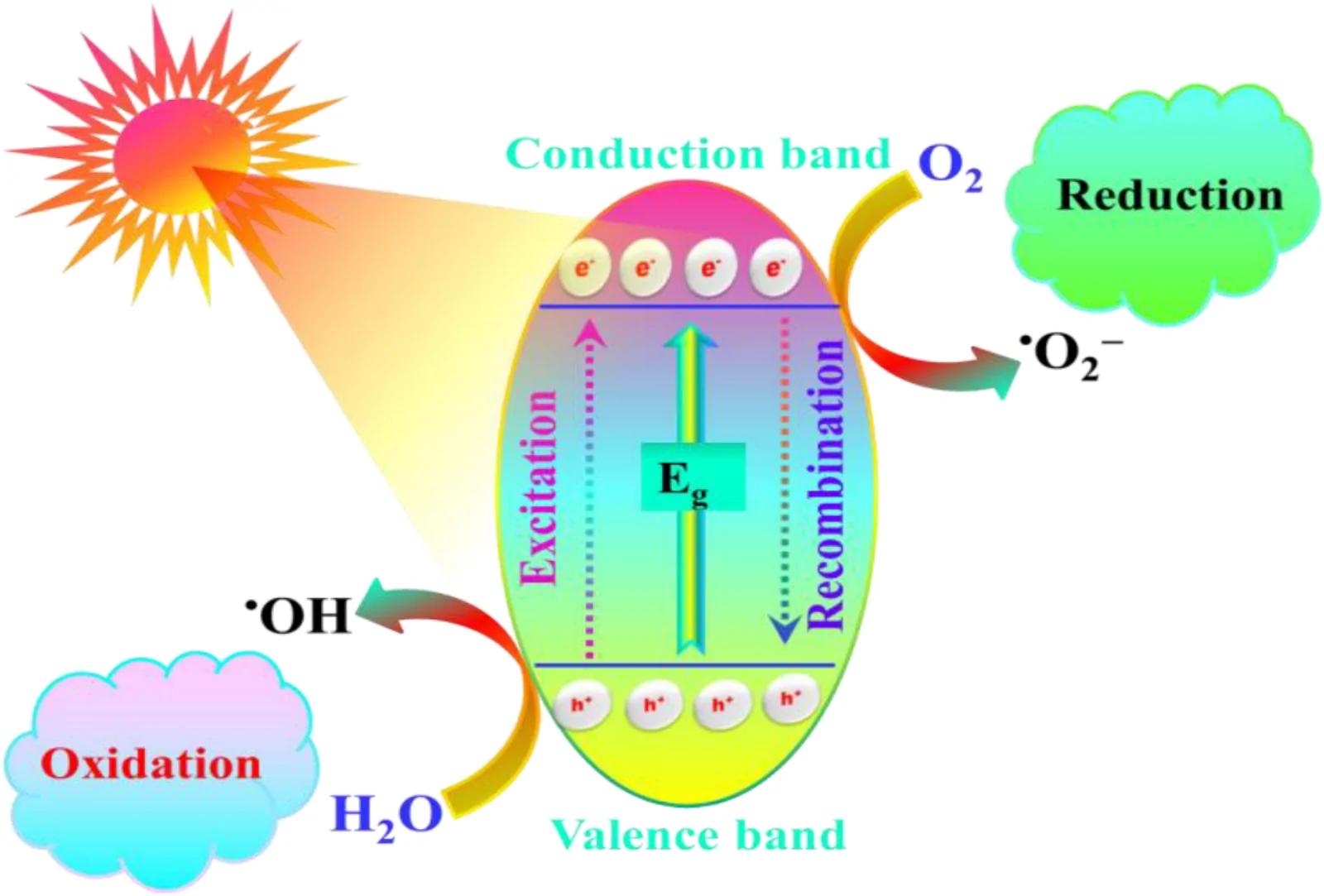
Schematic of photocatalytic mechanism in polymer-based systems showing light absorption, charge separation, migration, and surface redox reactions. Reprinted with permission from ref. 14*Nanomaterial*, copyright 2024 under the Creative Commons attribution license.^19^

### 2.1 Exciton dynamics and electronic structure in polymer photocatalysts

Careful distinction between molecular orbital concepts (HOMO and LUMO) and the conventional solid-state band concepts, valence band (VB) and conduction band (CB), is needed to provide the photophysical description of polymer photocatalysts. HOMO and LUMO are the frontier molecular orbitals of single polymer chains, and the HOMO–LUMO gap corresponds to the molecular equivalent of the bandgap.^15^ Electronic states in periodic solids, however, in extended solids produced by the process of delocalizing the orbit in a unit cell give rise to extended electronic states and crystal momentum-dependent charges with transport that is band-like. If the polymer is highly ordered and crystalline, and has a high degree of π–π stacking, the difference is less distinct because the molecular orbitals mix to form extended states.^16^ But this band picture is a great approximation in poorly ordered or amorphous polymers (which are the majority of photocatalytic materials). In these systems, the electronic states are localized or slightly delocalized, and charge transport is hopping from sites of localized states rather than band conduction. The terms valence band and conduction band are used here specifically for the Type-II/Z-scheme/S-scheme classification framework (Section 2.2) and for highly crystalline components such as g-C_3_N_4_ and COFs, while HOMO/LUMO is used to describe frontier molecular orbitals for polymer-specific charge-transfer energetics.^17^ The absorption of a photon having energy higher than the HOMO–LUMO gap produces an exciton in the form of a bound electron–hole pair in photoactive materials. Due to low dielectric constants of organic polymers (*ε* ≈ 2–4), the Coulombic screening is weak, which leads to large exciton binding energies in the range 0.2–0.7 eV, as listed in Table 1. On the other hand, in case of inorganic semiconductors which offer higher dielectric constants (*ε* > 10), the attraction is strongly screened, which leads to a lower exciton binding energy is lower, typically having values less than 0.05 eV. Accordingly, in the case of polymers, the excitons actively dissociate into free charge carriers before their reaction takes place at the surface, and for several inorganic semiconductors, thermal energy often appears sufficient to cause the dissociation.^18^ For polymers, the exciton diffusion length (LD) is generally between 5 and 20 nm and depends on the ordering of the molecules and the crystallinity of the polymer. Depending on the degree of π–π stacking, it becomes more possible for excitons to migrate through these ordered areas, and less possible for energy to migrate through amorphous areas with higher energetic disorder and trapping sites. Exciton dissociation at the interfaces with driving force, such as donor–acceptor junctions, polymer–inorganic heterojunctions, or defect sites, is highly efficient, dictated by the exciton binding energy relative to the thermal energy (*k*_B_*T*) and the strength of the electric field at the interface.^19^

**Table 1:** Summary of charge dynamics in various polymer-based photocatalysts

Polymer type	Exciton binding energy (eV)	Charge mobility (cm^2^ V^−1^ s^−1^)	Exciton lifetime (ns)	Recombination rate	Key qualification	Ref.
Covalent organic frameworks	∼0.2–0.5	10^−3^–10^−1^	50–500	Low	Highly dependent on framework crystallinity and pore structure	24
Metal-free (g-C_3_N_4_)	∼0.3–0.6	10^−5^–10^−4^	0.5–5	Moderate	Sensitive to synthesis temperature and crystallinity	25
Conjugated (D–A)	∼0.2–0.4	10^−3^–10^−2^	10–100	Moderate	Strongly dependent on D–A ratio and film morphology	26
Conjugated microporous polymers	∼0.3–0.7	10^−4^–10^−2^	10–100	Moderate	Affected by crosslinking density and surface area	6
Conducting (PANI)	∼0.5	10^−4^–10^−3^	1–10	High	Values depend on doping level and protonation state	27

A bound electron–hole pair, with the electron situated on the acceptor and the hole on the donor, can be an intermediate state between the exciton and free charges, called a charge-transfer (CT) state. If it is stabilized by strong electronic coupling or favorable energetics, it dissociates into free carriers, otherwise, it recombines geminately.^20^ Recombination can take place *via* radiative (fluorescence/phosphorescence), non-radiative (heat dissipation), geminate (back to original pair recombination) or bimolecular (free electrons and free holes from different excitations).^21^ The charge transfer from the polymer to co-catalysts, inorganic semiconductors and to the reactants is controlled by four factors including, energy level alignment, the energy level of the LUMO in the polymer needs to be more negative than the reduction potential of the acceptor. The HOMO energy level in the polymer needs to be more positive than the oxidation potential of the donor. Electronic coupling must be strong enough to facilitate the transfer of charge. Interface electric fields which is band bending or dipole layers at the interface must facilitate the transfer of charge in a certain direction. However, interface defects can act as channels for charge transfer or recombination centers.^22^ There are both intrinsic (crystallinity, molecular weight, defect states) and extrinsic (temperature, solvent/electrolyte, electron donors/acceptors) factors that affect charge separation and migration efficiency.^23^ Quantitative comparison of exciton binding energies, charge mobilities, lifetimes, and recombination rates for various polymer classes is summarized in Table 1, which shows variations from polymer to polymer that are particularly critical for photocatalytic performance.

### 2.2 Heterojunction mechanisms and their experimental validations

There are three main charge transfer mechanisms responsible for the improvement in photocatalytic activity in a polymer-based heterojunction, namely Type-II, Z-scheme, and step-scheme (S-scheme) mechanisms.^28^ In Type-II heterojunctions, a photogenerated electron will be transferred from the higher to the lower conduction band minima (CBM), while a hole will be transferred from the lower to the higher valence band maxima (VBM), with a loss of redox potential in both cases but a gain in spatial charge separation.^29^ The Z-scheme systems are designed to mimic natural photosynthesis, in which electrons released from the more negative CBM recombine with holes from the less positive VBM *via* an interfacial pathway, leaving the strongest reducing/oxidizing species intact. The S-scheme extends this idea by introducing more stringent thermodynamic criteria that involve recombination of electrons from the semiconductor, which are of the reduction type, with holes, which are of the oxidation type, under the influence of the internal electric field, which is determined by the differences in work function.^30^ A critical evaluation shows that assignments to mechanisms in the literature are often based on inadequate evidence. It must be noted that a reduction in the photoluminescence does not guarantee a specific mechanism, as quenching can occur *via* non-radiative recombination, *via* energy transfer or *via* trapping of defects at the interface, rather than by charge transfer across the interface.^28^ However, thermodynamic feasibility has been suggested by band alignments but cannot provide evidence for the actual pathway(s) of charge transfer. The increased photocatalytic activity can only be attributed to one mechanism if direct evidence is found, as there are several factors that affect the photocatalytic activity.^31^

Complementary techniques are required to assign the mechanism, which include: Kelvin Probe Force Microscopy (KPFM) work function mapping; Ultraviolet Photoelectron Spectroscopy (UPS) for VBM and work function; X-ray Photoelectron Spectroscopy (XPS) core level binding energy shifts; *in situ* XPS for surface potential changes under illumination; Transient Absorption Spectroscopy (TAS) charge transfer dynamics; and Surface Photo voltage Spectroscopy (SPS) surface potential changes under illumination.^32^ There are important discrepancies in the literature. The reported mechanisms (Type-II, Z-scheme, S-scheme) often vary for the same material system, usually reflecting slight differences in the way they are synthesized, or the type of characterization technique they are measured with, and not actual differences in their mechanism.^33^ Despite the possible non-specific reactions and possible trapping agent influence on the reaction environment, many studies over interpret the results of radical trapping for the assignment of a Z-scheme or S-scheme. The direction of the internal electric field is left unestablished, and the work function measurements are often ignored, which is essential to understand whether it is a Z-scheme or an S-scheme.^34^ In the majority of mechanistic studies, the characterization of individual components is used to make assignments, and there is a lack of *in situ* or *operando* studies. At least three complementary techniques of different types (band structure determination, photophysical studies, radical detection, and *in situ* characterization) should be used, with different types indicated. When a Z-scheme or S-scheme mechanism is proposed, work function measurements (UPS or KPFM) should be added. It is recommended that charge transfer kinetics be monitored directly with transient absorption spectroscopy.^35^Table 2 summarizes the synthesis routes used to fabricate the polymer-based heterojunctions as well as relevant literature which reports the confirmation of interfacial charge transfer, instead of mere band alignment or successful synthesis.

**Table 2:** Fabrication routes for polymer-based heterojunctions, and experimental evidence of interfacial charge transfer

Technique	Thickness	Time	Advantages	Limitations	Charge-transfer	Ref.
CVD	1–100 nm	Hours	Uniform; precise	High temp; expensive	Type-I *vs.* Type-II confirmed	36
ALD	<1 nm per cycle	Hours to days	Atomic precision; uniform	High temp; slow	—	37
Co-catalyst deposition	0.1–5 wt%	Hours	Charge separation; kinetics	Noble metals; aggregation	p–n junction confirmed	38
Dye sensitization	Monolayer	Hours	Vis-light absorption; low cost	Stability; photobleaching	h^+^ transfer confirmed	39
Chemical grafting	1–50 nm	Hours	Covalent; stable	Complex; limited surfaces	Z-scheme confirmed	40
Dip-coating	10–500 nm	Min to hours	Simple; scalable; low cost	Thickness control difficult	Type-II	41
Spin-coating	10–1000 nm	Minutes	Fast; uniform	Material waste; flat substrates	—	42
Electrospinning	100 nm–10 µm	Hours	High surface area; nanofibers	Cost; parameter optimization	Confirmed *via in situ* XPS	43
Sol–gel coating	10–1000 nm	Hours	Versatile; low temp	Shrinkage; cracking	S-scheme confirmed	44
Photo-deposition	0.1–5 wt%	Min to hours	Selective; RT	Photoactive materials only	Charge-transfer pathway confirmed	45

## 3 Synthesis techniques

The polymer-based photocatalysts are prepared by a variety of experimental strategies, of which a few are described below.

### 3.1 Chemical synthesis approach

Coupling reactions, involving appropriate functional groups on the aromatic and heteroaromatic monomers, are frequently used to synthesize the conjugated polymers. In the resulting polymers, the pi-electrons can be extended in the molecule to absorb visible light and to transport charge. The synthesis of g-C_3_N_4_ is generally performed by the thermal condensation of nitrogen-rich precursors, such as urea, thiourea, melamine or cyanamide, at high temperature. This process leads to a layered polymeric material with photocatalytic activity.^46^ COFs are synthesized through the controlled condensation reactions of organic building blocks with specific symmetries. The choice of monomers and reaction conditions allows for the creation of crystalline porous polymers with tailored pore structures and functionalities for photocatalysis.^47^ For the preparation of photoactive redox polymer films, the electropolymerization method is used to obtain an imidazole group. Moreover, photoactive porous organic polymers (POP) is obtained directly, which are dicyanobenzene-carbazole dye-functionalized.^48^

### 3.2 Nano-structuring and morphology control

To achieve the optimal photocatalytic effect, important aspects are nanostructuring and morphology control strategies, which will enhance the surface area, light absorption, charge transport, and accessibility of the active sites.^49^ As illustrated in Fig. 3, the 2D polymer heterojunctions are fabricated strategically through template-assisted methods, by first synthesizing individual 2D polymer sheets with different polymer 2D morphologies as structural matrices, followed by complementary 2D polymer deposition by template-assisted strategies. The interface between the donor and the acceptor is an intimate layered junction that creates a sharp donor/acceptor heterojunction interface aimed at achieving maximum exciton dissociation and precise directional charge separation. Lastly, the acceptor surface is decorated with Pt nanoparticles and single-atom co-catalysts by controlled photodeposition, which is crucial to improve the surface catalytic kinetics in producing H_2_ with greatly higher efficiency.

**Fig. 3:**
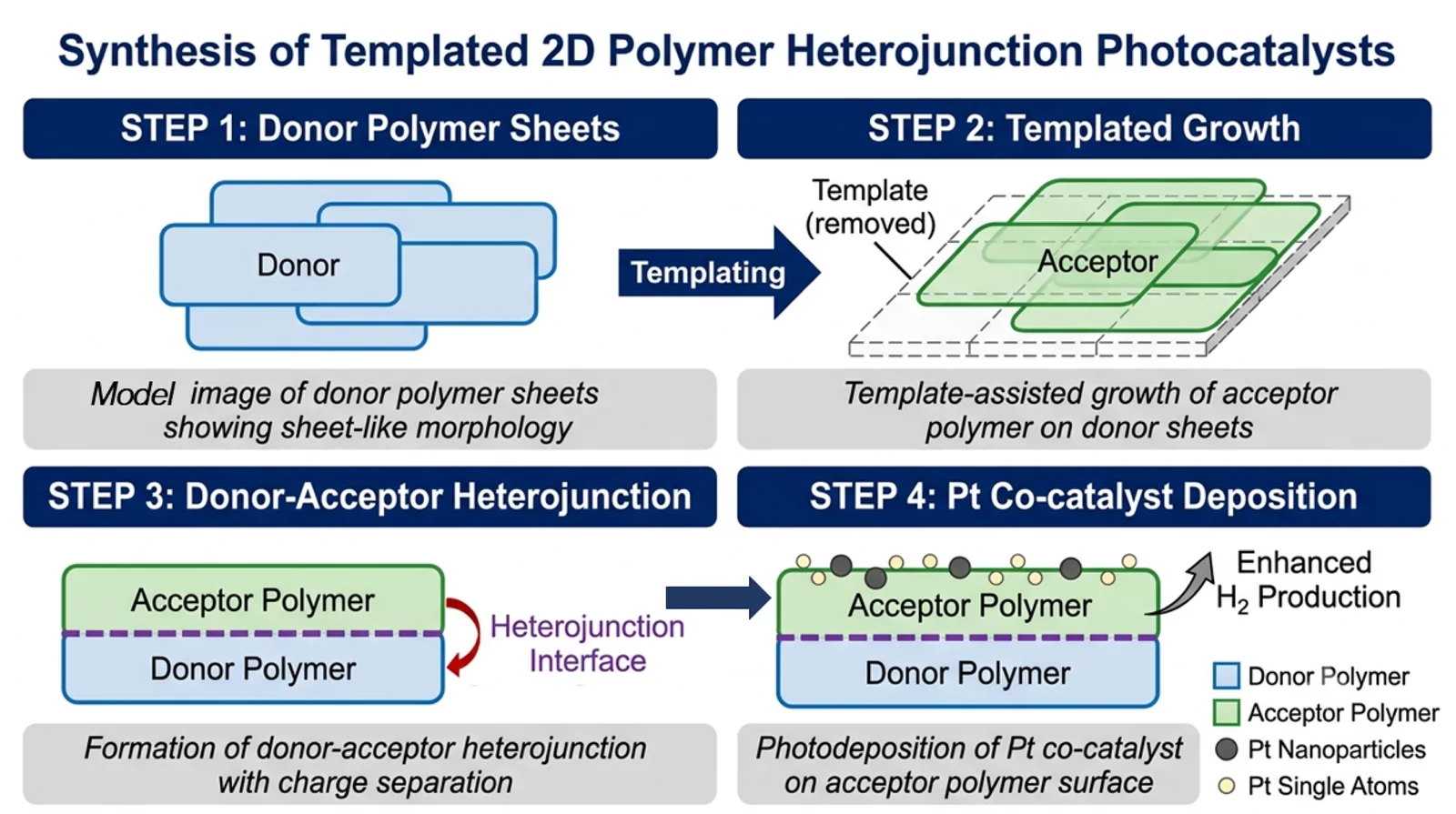
Schematic illustration of the synthesis (donor sheets → templated growth → heterojunction formation) and Pt co-catalyst modification of 2D polymer heterojunctions for enhanced H_2_ production.

The ability to hydrogen bond and π–π stacking orders polymers into ordered nanostructures.^26^ Thin-film self-assembly processes are performed on the inner surface, and organized nanostructures are produced. Immiscible phase boundaries were considered in ref. 31. Living Radical Polymerization (LRP) techniques like atom transfer radical polymerization (ATRP), reversible addition–fragmentation chain transfer (RAFT), and nitroxide-mediated polymerization (NMP) processes enable control of the molecular weight and architecture of the resultant polymer chains.^32^ This control can indirectly affect the self-assembly behaviour and the resulting nanostructure. As an example, block copolymers prepared by LRP are able to self-assemble to well-defined micelles or other nanophases.^33^

The electrospinning is one of such methods that a polymer solution is ejected from a spinneret under a high electric field.^34^ Due to electrostatic forces, the solution is stretched into continuous nanofibers, and different parameters, such as voltage, flow rate and solution concentration, can be used to obtain nanofibers with different diameters and orientations. The method for synthesis controlled size and shape of polymer nanoparticles is the method of emulsion polymerization or precipitation polymerization.^35^ The higher order nanostructures can then be created by methods such as layer-by-layer assembly or directed self-assembly of these nanoparticles. Polymer thin films can be directly patterned into desired nanoscale features by lithography-based techniques like nanolithography.^36^ The nucleation and growth steps are very sensitive to the nature of the polymerization parameters, including temperature, reaction time, concentration of the monomers, nature of the solvent, and additives used (such as capping agents and stabilizers) and can be used to control the size and shape of the resulting polymer nanostructures.^37^ These approaches can be strategically used individually or in combination to control the nanostructure and morphology of polymer photocatalysts, leading to the control of light absorption, charge separation, charge transport and surface reactivity for the most appropriate application, thereby improving the photocatalytic efficiency of the material.

### 3.3 Surface modification and functionalization

In terms of post-synthetic methods, surface modification and functionalization are involved in the chemical modification of the interface between polymeric photocatalysts and the target substances. It helps achieve maximum light-harvesting efficiency, long-term chemical stability, and affinity toward the target substance, while maintaining the chemical selectivity of charge transfer.^38^ These interfacial modifications are usually carried out after the basic polymerization and morphological optimization, and they have a profound influence on the interaction of the catalyst with the reaction medium. Macromolecular grafting is one of the most powerful strategies to covalently or non-covalently attach functional molecules or additional polymeric units to the main photocatalytic backbone.^39^ This process usually begins with the activation of functional groups on the surface (often accomplished by generating radicals) and involves the coupling of the functionalizing agent. There are two general categories of grafting topologies: grafting-from and grafting-through. The grafting-from method is based on immobilization of molecular initiators directly on the polymer photocatalyst surface, and the polymerization *in situ* of additional monomer units. This configuration enables control of the resulting graft density, spatial orientation and chain length.^40^ On the other hand, the grafting-through mechanism refers to the direct copolymerization of the main macromolecular precursor and a second functional monomer. Thus, the desired functional groups are incorporated as part of the structure during chain propagation, and thus their distribution is segregated to or enriched in the interfacial boundary layer according to the relative reactivity of the monomers and thermodynamic curing conditions.^41^

In addition to grafting, some of the most developed surface coating strategies are advanced surface coating techniques, such as layer-by-layer (LbL) self-assembly. The method is based on the sequential electrostatic adsorption of oppositely charged species, including photoactive functional molecules, catalytic nanoparticles, and co-catalytic ones, to the polymeric scaffold, which leads to highly ordered and multi-functional thin-film architectures whose interfacial kinetic properties are larger than those of the constituents.^42^ The electrostatic interactions drive the formation of a multilayered coating with controlled thickness and composition, allowing for the introduction of specific functionalities.

Chemical modification of surface functional groups involves reacting existing functional groups on the polymer surface to introduce new functionalities. Such as hydrolysis or esterification, as modifying ester or amide groups on the polymer surface. Amination or amidation is the introduction or modification of amine or amide functionalities. Sulfonation or oxidation introduces sulfur-containing or oxygen-containing groups. Using highly efficient and selective reactions (*e.g.*, copper-catalyzed azide–alkyne cycloaddition) to attach functional molecules to the surface.^50^ For enhanced photocatalytic activity, especially for reactions like water splitting or CO_2_ reduction, co-catalysts (often metal nanoparticles or metal oxides) can be selectively deposited onto the surface of the polymer photocatalyst.^51^ This can be achieved through photoreduction of metal precursors, chemical reduction, or deposition–precipitation methods. The co-catalysts facilitate charge separation and surface reaction kinetics. Organic or inorganic dye molecules can be adsorbed or covalently attached to the surface of the polymer photocatalyst to extend its light absorption range into the visible region.^52^ The dye absorbs light and injects electrons into the LUMO of the polymer, initiating the photocatalytic process.

A suitable surface modification technique is needed to gain the desired photocatalytic performance. Table 3 shows the results of comparison and mechanism of the most commonly used surface modification techniques. These surface modification and functionalization strategies can be used to optimize the properties of polymer photocatalysts for various photocatalytic applications, leading to more efficient, selective, and stable photocatalysts. The choice of method depends on the desired functionality, the nature of the polymer photocatalyst, and the specific application requirements.

**Table 3:** Comparison of surface modification techniques for polymer photocatalysts

Technique	Film thickness	Modification time	Key advantages	Main limitations	References
Chemical vapor deposition (CVD)	1–100 nm	Hours	Uniform coverage, precise thickness	High temperature, expensive equipment	53
Atomic layer deposition (ALD)	<1 nm per cycle	Hours to days	Atomic precision, excellent uniformity	High temperature needed	54
Layer-by-layer (LbL) assembly	1–100 nm (per layer: 1–10 nm)	Hours to days	Precise thickness control	Time-consuming, requires charged species	55
Co-catalyst deposition	0.1–5 wt% loading	Hours	Enhanced charge separation, improved kinetics	Noble metals are expensive, aggregation is possible	56
Dye sensitization	Monolayer (0.01–0.5 molecules per nm^2^)	Hours	Visible light absorption, low cost	Dye stability issues, photobleaching	57

### 3.4 Green and scalable synthesis

Yielding unique morphologies, microwave-assisted synthesis is an effective technique that improves charge separation by shortening polymerization or condensation times with exfoliation and defect formation. The best photocatalytic activity is observed for 30 wt% g-C_3_N_4_/TiO_2_. It degrades MO dye in 4 hours with 85% efficiency. This efficiency is much larger than that of the pristine structure.^58^ A seed-induced solvothermal process at 105 °C uses visible light and degrades MO in 60 minutes with 100% efficiency. It utilizes g-C_3_N_4_/TiO_2_ mesoporous material, and the reported kinetic constant is 0.1014 per min.^59^ 97% MO degradation is investigated by g-C_3_N_4_ (nanosheets)/TiO_2_ (nanoflakes) with 0.0718 per min constant value. The *in situ* sol–gel method is applied at 400 °C. 20 minutes, exposing of g-C_3_N_4_/TiO_2_ nanotube array, which degrades ∼85% MO.^60^ Moreover, the energy gap decreases when the temperature increases. At 550 °C and 450 °C the gap observed for g-C_3_N_4_ is 2.66 eV and 2.82 eV, respectively.^61^ It is a very fast process, improves the dispersion, and undergoes doping that overall affects the morphology of the materials. As it is directly related to the temperature, careful and controllable conditions for temperature conditions are important. The temperature affects the material effectively, which can change the overall output.

All the discussed techniques are beneficial as they provide eco-friendly, energy-efficient, and rapid solutions. Table 4 compares the performance metrics of various synthesis techniques commonly used for the preparation of polymer photocatalysts. The advanced techniques could control morphology and porosity more effectively than conventional strategies. Photocatalytic activities in applications for survival by dealing with environmental and energy hazards improve many important phenomena. To make polymer-based photocatalysts industrialized, research in this specific area is essential, with a brief mechanistic activity.

**Table 4:** A quantitative comparison of the performance merits of various synthesis techniques for polymer photocatalysts

Synthesis method	Particle size (nm)	Surface area (m^2^ g^−1^)	Synthesis time	Yield (%)	Scalability	Energy consumption	Ref.
Chemical (Suzuki coupling)	100–1000 (bulk)	10–100	24–72 h	40–80	Moderate	High	62
Thermal polymerization	500–5000	20–90	2–6 h	60–95	High	Very high	63
Nanoprecipitation	5–100	50–200	5–30 min	70–95	High	Low	64
Miniemulsion	50–500	30–150	2–12 h	60–90	High	Moderate	65
Template synthesis	10–500	100–1000	12–48 h	40–80	Low	Moderate	66
Microwave-assisted	10–100	50–300	10–60 min	70–95	Moderate	Moderate	67
Ultrasound-assisted	20–200	100–500	10–60 min	60–90	Low	Low	68
Solvent-free	1000–10000	10–50	2–12 h	80–95	High	High	69

### 3.5 Strategies for enhanced photocatalytic activity

All of these classes of polymer suffer from a common drawback in which pristine conjugated polymers have low photocatalytic activity owing to the fast recombination of electron–hole pairs. There are several intrinsic limitations: high exciton binding energy that prevents charge separation, low charge carrier mobility in the amorphous part of the material, and the absence of an intrinsic driving force for the separation of charges in space. To overcome these limitations, there are three major approaches that have been developed: elemental doping, heterojunction formation, and surface modification.^70,71^

Elemental doping involves incorporation of heteroatoms into the polymer structure to alter its electronic properties. The effect can be explained with the help of the prototype of bromine-doped polymeric carbon nitride (Br–PCN). The high planar symmetry of pristine PCN gives the HOMO and LUMO a uniform delocalization over the tri-*s*-triazine rings, as shown by the density functional theory (DFT) calculations. When bromine atoms are incorporated, the bromine atom has a larger atomic radius than the original atoms, resulting in distortion of the original planar structure. The structural distortion gives rise to spatial separation of HOMO and LUMO; spatial redistribution of the orbitals on a conjugate region enhances exciton dissociation and leads to redistribution of electrons and holes in the corresponding units.^70,71^ Bromine doping redistributes charge and enhances electrical conductivity. Together, these effects promote continuous charge transfer and minimize recombination. The doped bromine is also a promoter for electrical conductivity which has a beneficial effect for charge separation.^70^ Experimental results also reveal that the band gap of g-C_3_N_4_ is reduced in the presence of bromine doping and the absorption of light is increased with a significant improvement in the photocatalytic activity. For instance, the uranium reduction efficiency increased 2.2 times with good stability after five cycles.^72^

An alternative and complementary approach to reducing recombination is to form a heterojunction. If two semiconductors with complementary band structures are placed in contact, a built-in electric field is created at the interface, which results in the directionality of the charge migration. Poly(3-hexylthiophene) (P3HT) can be used as a suitable model compound. Despite the high ability of carrier transport and the broad absorption range (band gap 1.9–2.1 eV) as well as the hole mobility that is comparable to amorphous silicon, the pristine form of P3HT has almost no photocatalytic activity because of the short distance of the carrier migration and the fast electron–hole recombination speed.^73^ However, π–π interactions can be used to form well-defined p–n heterojunctions between P3HT and g-C_3_N_4_, which enables efficient separation of electron–hole pairs. The photocatalytic hydrogen evolution rate (HER) of the resulting P3HT/g-C_3_N_4_ composite fiber membranes is 7327 µmol (h^−1^ g^−1^), which is 57.7 times higher than that of the pristine P3HT fibers and 6.8 times higher than that of powder g-C_3_N_4_.^73^ The heterojunctions actually prevent the recombination of electron–hole pairs generated by the photons, and increase the quantum efficiency. Recent studies of D–A conjugated polymer/g-C_3_N_4_ heterojunctions also show that differences in electron density at the polymer's interface can lead to diffusion-controlled charge separation that can outweigh the effects of the internal electric field.^74^ There is a minor modification required in the donor–acceptor structure to change the charge separation mechanism from Type-I to Type-II. The heterojunction between the benzothiadiazole-thiophene copolymer (BSO-T) and graphitic carbon nitride (GCN) shows an enhanced HER of 19.36 mmol g^−1^ h^−1^, which is 13.6-fold and 7.0-fold higher than those of GCN and BSO-T alone, respectively, due to the effective diffusion-controlled charge separation.^74^

The third strategy is surface modification. Functional groups or co-catalysts when introduced to the polymer surface, create reaction sites that act as a means to remove the photogenerated carriers before they can recombine. For P3HT/g-C_3_N_4_ systems, the selection of the sacrificial reagent can significantly impact the photoactivity; for example, the H_2_ evolution activity for the surface heterojunction catalyst with 3 wt% P3HT is up to 3045 µmol h^−1^ in saturated ascorbic acid (AA) solution, which is about 491-fold higher than in the absence of AA under light irradiation of *λ* ≥ 500 nm.^73^ The same system achieves a record AQY of 77.4% at 420 nm, with broad visible/NIR-light responsiveness, showing apparent quantum yield AQY values of 59.4%, 20.2%, 3.2%, and 0.68% at 500, 600, 700, and 800 nm, respectively.^73^ The very high photoactivity is due to the characteristics of the low threshold for absorption of visible light, high efficiency of the charge transfer at the polymer/polymer interface and the appropriate oxidation half-reaction made possible by the use of the sacrificial reagent.

The difference between these strategies is not only theoretical, as the doping changes the intrinsic electronic structure of the polymer, while the heterojunction brings about an interfacial driving force for charge separation, and the surface modification changes the rate of consumption of carriers. In practice, often these strategies are mixed. For instance, the synergistic effect of vacancy engineering and S-scheme charge transfer has been demonstrated in S-scheme S-C_3_N_4_/Bi_2_WO_6_ heterojunctions with interfacial oxygen and nitrogen dual vacancies, leading to reduced recombination and improved photocatalytic activity.^70,71,75^ These strategies can be combined to create a versatile package to deal with the native recombination deficiency of pure polymers.

#### 3.5.1 Contradictions and agreements for enhancement strategies

Three points of contention emerge from the discussion. First, the prevailing charge-separation process in polymer heterojunctions remains unresolved. The overall concept behind heterojunctions is to create an electric field at the interface. It has been demonstrated that diffusion-controlled separation is more important than field effects in donor–acceptor systems.^74^ Both mechanisms may operate, with their relative weight depending on interface quality and band offset. Second, fold-change is not a reliable performance metric on its own. Superiority is not really demonstrated by a large fold-change over a weak baseline. The 57.7-fold enhancement of P3HT/g-C_3_N_4_ reflects the near-zero pristine activity of P3HT, not the intrinsic merit of the heterojunction. The 6.8-fold gain over the stronger baseline, g-C_3_N_4_, is more informative.^73^ The same applies to BSO-T/GCN as the 7.0-fold gain over BSO-T isolates the heterojunction's contribution better than the 13.6-fold gain over GCN.^74^ The 491-fold gain from ascorbic acid addition is similarly inflated by a near-zero baseline; the absolute rate (3045 µmol h^−1^) and AQY (77.4% at 420 nm) are more reliable.^73^ Third, direct evidence for the heterojunction's role is rarely provided for the systems discussed here. As noted in Section 2.2, assigning a charge-transfer mechanism requires techniques such as transient absorption spectroscopy or work-function mapping. Neither ref. 73 nor 74 reports such measurements as the both appeared to report fold-change alone. A fold-change is consistent with a heterojunction effect as it does not, by itself, prove one. Despite these open questions, three points of agreement are consistent across the literature reviewed here. Pristine conjugated polymers show low activity due to fast recombination. Both doping and heterojunction formation measurably improve charge separation. Combining strategies, as in S-C_3_N_4_/Bi_2_WO_6_ with dual vacancy engineering, outperforms any single strategy alone.^70,71,75^

### 3.6 Analysis of synthesis–performance–morphology relationship

It is observed from the previously discussed synthetic methods that there are a few significant methodological constraints that are important to consider for the interpretation of the reported results and comparison of photocatalytic performance among the studies. Reproducibility and reporting standards are always an issue, because many synthesis procedures do not provide enough detail to be reproduced, and important parameters such as cooling protocol, stirring conditions, aging time, and air control, *etc.*, can have a great impact on product properties, but are often not mentioned in the experimental sections studies reporting unusually high photocatalyst activity should be treated with caution. Such claims require detailed synthesis procedures and evidence of reproducibility across independent syntheses, because synthesis reporting in the literature is not standardized.^76^ Few synthesis processes have been carefully studied for industrial scale, as most demonstrated only at the lab scale (*e.g.*, <1 g batch size) have not proved to be scalable; template synthesis and microwave-assisted methods, although useful for obtaining the desired morphologies, have several drawbacks for the industrial scale, such as cost, throughput and batch-to-batch variability, while thermal polymerization is the most scalable process, but results in a broad molecular weight distribution and lacks architectural control.^77^ Another assumption is that increased surface area is always a positive sign of increased photocatalytic activity, which is not necessarily true, and there are several exceptions, including wettability, crystallinity, surface defect density, pore structure, *etc.*, where interaction between these factors is complex and is not studied systematically.^78^ Studies which states that increased activity leads to increased surface area, without taking into account the other factors, should be regarded with suspicion. The designation of a synthesis route as green or sustainable also has to be carefully justified: although many routes are considered environmentally friendly because the precursors are bio-derived, in general, these materials show lower photocatalytic activity, and it is unclear whether the environmental benefit derives solely from the bio-derived feedstock. This notion that metal-free is equal to environmentally friendly is especially troublesome because the preparation of many metal-free polymers involves hazardous starting materials and organic solvents.^79^ Lastly, contradictory reports have been found regarding the importance of particular morphologies on photocatalytic activity: although the nanosheet morphology was found to be beneficial in general for charge separation because of the reduced charge transport distances, some studies have suggested that the bulk or mesoporous morphology leads to better performance. The conflicting information in these reports may stem from protocol variations in synthesis, crystallinity, defect density, or measurement, making it very important to have a standard characterization protocol for the morphology of these materials and other parameters controlled comparative studies to exclude the influence of morphology on other considerations.^80^ Systematic comparative investigations are an important deficit to address, and future efforts should focus on careful, well-controlled investigations to gain trustworthy correlations between morphology and performance, and rational design of catalysts.^81^

## 4 Types of polymer-based photocatalysts

There are varieties of polymer-based photocatalysts, including conducting polymers, conjugated polymers, and metal-free polymers.

### 4.1 Conducting polymer

The use of conducting polymer photocatalysis (CP photocatalysis) makes use of the inherent optoelectronic properties of intrinsically conducting polymers (CPs) both independently as metal-free organic photocatalysts and as photosensitizing components of multi-component composite structures. The organic pollutants have been degraded with 87.3% efficiency over 120 minutes in the presence of visible light on hydrothermally synthesized BiPO_4_/PANI hybridization platforms.^67^ In the same way, electrospun PANI/BiOBr nanofibrous networks have been tested against Rhodamine B (RhB) and achieved a very high 99.9% decomposition efficiency in 75 minutes.^68^ Moreover, in PANI/BiOCl nanosheets synthesized by the chemisorption technique, the photocatalytic degradation of methyl orange in water was enhanced from 67% in 20 min.^69^ Additionally, nanosheets of PANI/Bi_12_O_17_C_12_ prepared *via* an *in situ* route showed exceptional degradation pathways for multiple pollutants, degrading 91% of the ciprofloxacin and 99% of Rhodamine B after 120 and 100 minutes, respectively, under a 300 W Xenon lamp simulating the entire solar spectrum.^70^

Different ratios of PANI/TiO_2_ photocatalysts have been investigated in the presence of UV and visible light for Rhodamine B dye as a pollutant. The conducting polymer, PANI is environmentally friendly and stable. As the concentration of PANI changes, the colour changes accordingly. Fig. 3 illustrates the maximum degradation for P25. The pristine polymer is non-functional. PPy-TiO_2_ degrades methyl orange in 60 minutes. A 500 W xenon lamp is used as a light source during its manufacturing process by polymerization. 100% efficiency of this photocatalyst is observed during its operation.^82^ Malachite green pollutant is degraded in 120 minutes with 97% efficiency by BiOBr-Ag-PPy photocatalysts, when a 150 W halogen lamp has been used as a source.^83^ A 100% efficient PPy/Bi_2_WO_6_ photocatalyst has been prepared by the photocatalytic oxidative polymerization method. When a 500 W xenon lamp has been used as a source of light, it degrades phenol in 120 minutes.^84^ ZnO-microrods/PPy with a 1.7 eV band gap degrade the methylene blue.^85^

Combining conducting polymers with TiO_2_ is thought to be a viable technique to create extremely effective non-metal-based photocatalysts. The easy wet incipient impregnation approach has been used to fabricate TiO_2_@PANI, TiO_2_@PPy, and TiO_2_@PEDOT photocatalyst heterostructures.^86^ The conducting polymer mass ratios in the composites were tuned. XRD, TEM, TGA, DRS, and N_2_-physisorption techniques have been used to assess the samples' structure, morphology, optical characteristics, and surface texture. The highest H_2_ evolution rate (HER) has been observed in the TiO_2_@2PEDOT, TiO_2_@2PPy, and TiO_2_@5PAn composites, with respective values of 1.37, 2.09, and 3.1 mmol h^−1^ g^−1^. The HER has been considerably increased by 16, 24, and 36 times, respectively, in comparison to bare TiO_2_.^87^

The composite powder becomes darker with the increase in the amount of PANI, as seen in Fig. 4(a), with a colour shift from the white of TiO_2_ to the near-black of PANI-ES, which corresponds to the longer π-conjugation in the backbone of the PANI. This compositional change significantly influences the degradation of RhB, as shown in Fig. 4(b). The composite with the lowest amount of PANI (PT1100) shows almost as good degradation efficiency as bare P25 within 60 min, while it becomes less complete in the PT150, PT110, PT11, PT101, and PT1001 series with increasing PANI content and effectively negligible degradation with pure PANI-ES. This indicates that there is a threshold limit for the loading of PANI that will not impede activity in form of small amount. However, higher amount will inhibit activity, probably either due to a chromophoric association with TiO_2_ that reduces activity or due to disrupting the adsorption–desorption equilibrium that was established in the dark pre-equilibration period by the RhB.

**Fig. 4:**
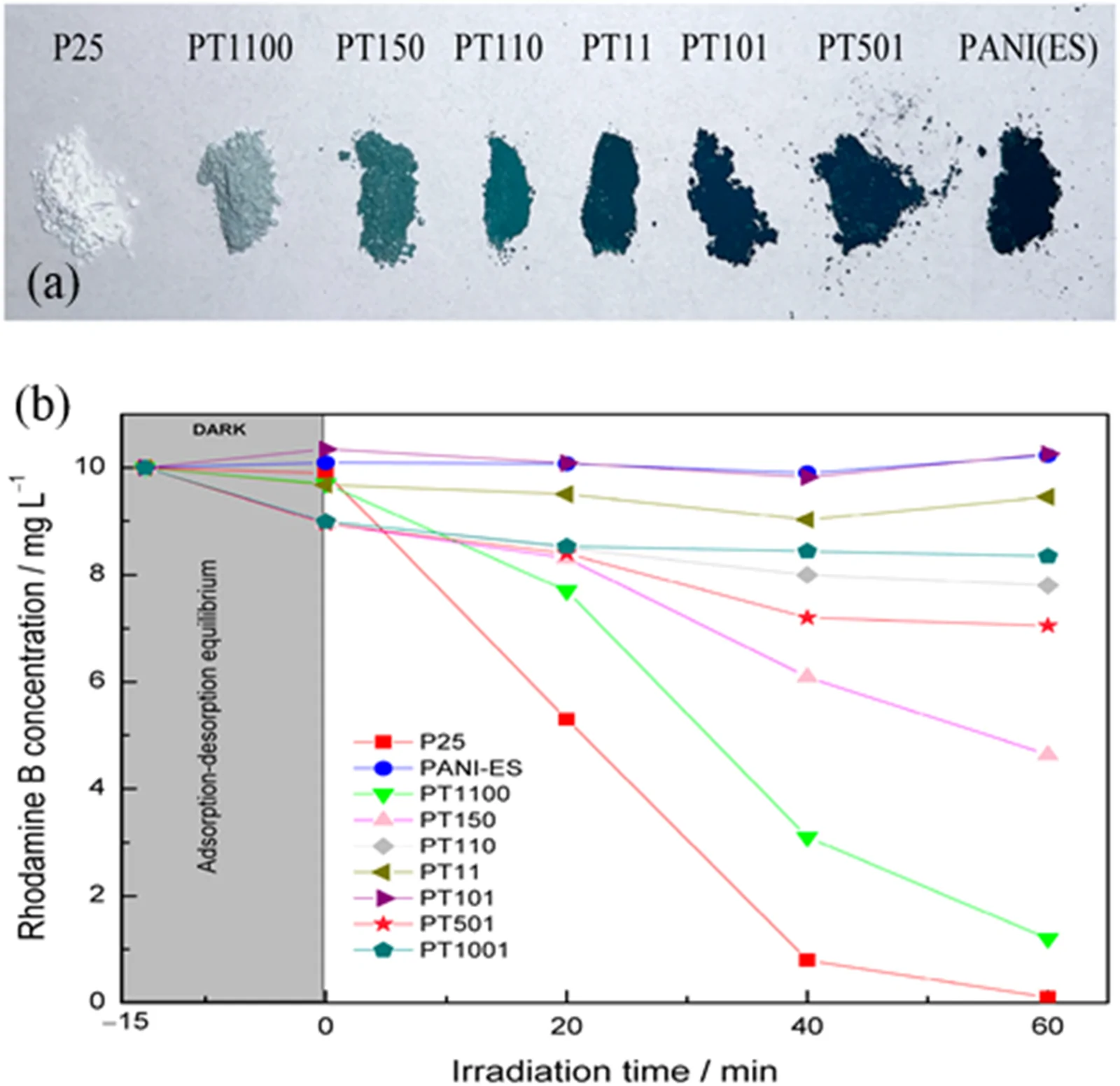
(a) Photographs of PANI/TiO_2_ composite powders (PT1100–PT1001), pristine TiO_2_ (P25), and PANI-ES, showing color darkening with increasing PANI content. (b) Photocatalytic degradation of Rhodamine B (RhB) under UV light for the same samples. Reprinted with permission from ref. 88.

This photocatalytic activity of PANI and PPy is related to the electronic structure of the conducting polymer, such as their conjugation and doping behaviour. These polymers contain single and double bonds between carbon atoms with unhybridized p_*z*_ electron clouds overlapping and resulting in high delocalization. This conjugated lattice can be modified by chemical or electrochemical doping, which results in defects and local deformations in form of polarons or bipolarons. These intermediate energy levels are far below the electronic energy gap, and the mobility of delocalised π-electrons along the polymer chain is high. Within composite photocatalysts, this high conductivity is a fast transport pathway which is important for it to not destructively recombine with charge carriers created in the attached inorganic semiconductor in the photocatalyst, like TiO_2_.

### 4.2 Conjugated polymers

A polymer–inorganic hybrid photocatalyst consists of a combination of both polymers (organic materials formed by long repeating chains of molecules) and inorganic materials (such as metal oxides or sulfides) to improve the effect of the photocatalyst in terms of using light to catalyze chemical reactions.^82^ A polymer that is combined with the light absorbing and catalytic properties of an inorganic material and has the flexibility and processability of a polymer. This mixture can produce a material with not only good photocatalytic activity, but also be more easily handled, more stable, and even more effective than its individual components. The inorganic part is normally the main light absorber, and it is expected to create electron–hole pairs upon illumination by light. The active charge carriers are then used to catalyze the desired chemical reactions, such as the reduction of pollutants or the generation of hydrogen. The polymer matrix may serve multiple important functions, such as dispersing inorganic nanoparticles, preventing them from aggregating with each other, giving mechanical strength and flexibility, helping to convey charges and improving the adsorption of reactants. A judicious selection of the polymer and the inorganic components and control over the interaction between them would allow the scientists to prepare hybrid photocatalysts with optimized properties for different applications, such as environmental remediation, energy conversion, and organic synthesis. *In situ* loading of cobalt and the presence of an electron scavenger, the first examples of linear conjugated organic polymer photocatalysts which produce oxygen from water were recorded.^83^

The thermodynamic driving force for water oxidation, the polymer's light absorption, and the aqueous dispersibility of the comparatively hydrophilic polymer particles all contributed to the faster oxygen evolution rates compared to similar organic materials. The best-performing system was further investigated using transient absorption spectroscopy, revealing that the presence of an electron scavenger minimizes recombination by facilitating rapid oxidative quenching of the exciton (electron–hole pair). H_2_ yield by photocatalytic method is observed for the donor–acceptor conjugated copolymers. The polymerization method forms heterojunctions which are polymeric–inorganic in nature. HER for pristine thianthrene dioxide (TSO_2_) is less than 1260 times comparable to the TSO_2_@TiO_2_; which is about 11 220 µmol g^−1^ h^−1^.^89^ The HOMO level of donor–acceptor TSO_2_ is less positive. TSO_2_ phase contain e^−^/h^+^ pairs while interacting the light, H_2_ gas production has been enabled by the generated built-in electric field at the junction between the two materials. TSO_2_@TiO_2_ reveal the improved H_2_ gas production by the efficient charge separation.

Donor–acceptor conjugated copolymers have been used for the investigation of photocatalytic hydrogen evolution (PHE) for water splitting. 1.618 mmol g^−1^ h^−1^ hydrogen evolution activity is investigated for T-CN-0.010 photocatalysts on g-C_3_N_4_.^90^ At 420 nm, 4.8% quantum efficiency has been observed. The photoresponse for ultrathin crystalline g-C_3_N_4_ nanosheets (UCCN) is about 74% at 420 nm, which yields 9.7 mmol g^−1^ h^−1^ hydrogen evolution when a light of more than 420 nm has been incident.^91^ The achievable light-to-energy conversion efficiency for the sulfur-doped carbon nitride (CNSO-20) photocatalyst has been approximately 11% with a rate of 5.02 mmol g^−1^ h^−1^.^92^ COF-based donor–acceptor polymers as photocatalysts involve H_2_ evolution with 6.9% photoresponse observed for the triazine-based covalent organic framework (Tz-COF-3) photocatalyst, which evolves 43.2 mmol g^−1^ h^−1^ hydrogen.^93^ Zinc porphyrin-tetrathiafulvalene covalent organic framework (Zn-por-TT COF) evolves 8.2 mmol g^−1^ h^−1^ hydrogen. Benzo[*b*]thiophene-2-carboxylic acid-based photocatalyst (BTH-3) exhibits a 1.25% photon conversion rate with 15.1 mmol g^−1^ h^−1^ hydrogen activity.^94^ At 450 nm, about 83% quantum efficiency has been noted for cyano-functionalized covalent organic framework (CYANO-COF), in which a 300 W xenon lamp has been used as a light source with 60.85 mmol g^−1^ h^−1^ hydrogen evolution rate.^95^

Sulfur-doped polyimide (SPI) with a series of zinc–sulfide polymer photocatalysts has been prepared by the solvothermal synthesis.^96^ The hydrogen production rate by photocatalytic phenomena has been observed to be highest for SPI composite/10ZnS composite. ∼2.8 time's higher rate has been observed with a ∼217 µmol g^−1^ h^−1^ value, indicating enhanced charge separation and activity with Z-type heterojunctions that ultimately increase the contact SPI and ZNS nanoparticles. Fig. 5 demonstrates an experimental validation of Type-II and Z-scheme heterojunctions. The Z-scheme enhances the overall efficiency by the mutual coupling of the generation of superoxide and hydroxyl radicals by the reduction and oxidation, respectively. In addition, steady state and time-resolved photoluminescence analysis revealed strong peaks at 475 nm for SPI and weakest for ZnS. The improved charge carrier separation has been confirmed for the composite made with decreased emission intensity peaks. The future direction of conjugated polymers in the context of heterojunction photocatalysis is aimed at exploring new architectures which are capable to operate under continuous light exposure for extended periods of time, and are also stimuli responsive and oxygen resistant.

**Fig. 5:**
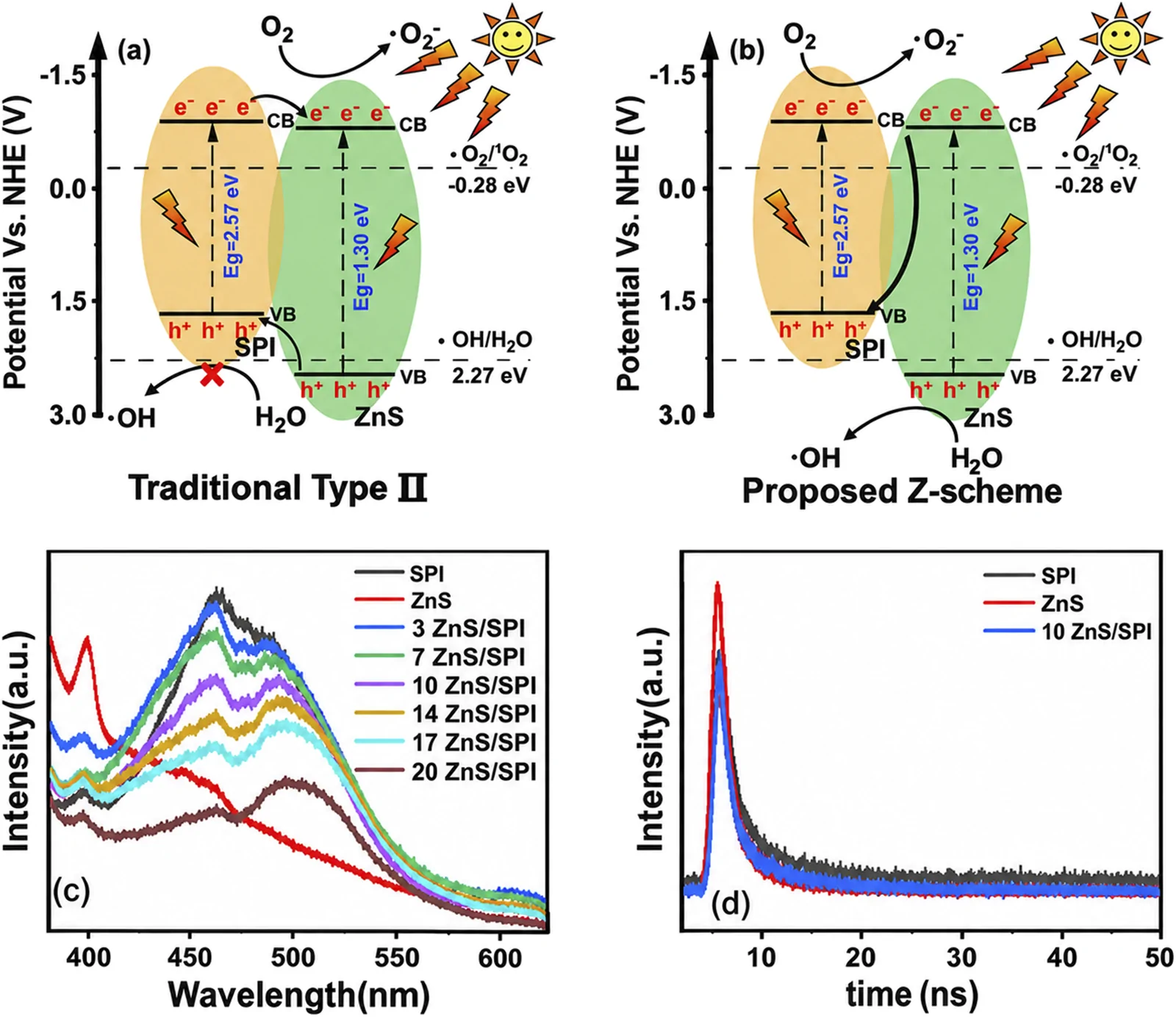
(a) Schematic illustration of the traditional Type-II charge transfer mechanism for the SPI/ZnS composite. (b) Proposed Z-scheme charge transfer mechanism. (c) Steady-state photoluminescence (PL) spectra and (d) time-resolved photoluminescence (TRPL) decay spectra of pristine SPI, pristine ZnS, and the 10ZnS/SPI composite. Reprinted with permission from ref. 96.

### 4.3 Metal-free polymer-based catalyst

A metal-free polymer photocatalyst is a type of photocatalytic material that utilizes polymers composed solely of organic elements (like carbon, nitrogen, oxygen, sulfur, *etc.*) to drive chemical reactions using light, without the inclusion of any metal-containing components.^97^ There are three relevant but often-conflated categories related to polymers: firstly, a metal-free polymer matrix, in which the polymer backbone itself contains no metal atoms (*e.g.*, pristine g-C_3_N_4_, COFs, or POPs); secondly a polymer/inorganic hybrid, in which a metal-free polymer is combined with a metal-containing co-catalyst or nanoparticle to enhance performance; and third one is a metal-containing heterojunction or framework, in which a metal is an intrinsic structural component rather than an additive (*e.g.*, a metal–organic framework or metal-doped covalent organic polymer) the examples discussed further are labeled accordingly. The components in form of a photocatalyst and a polymer are explained here. A material that accelerates a chemical reaction when exposed to light absorbs photons, which excite electrons and create electron–hole pairs. These energetic charge carriers can then initiate chemical transformations on the surface of the catalyst. Moreover, a large molecule is made up of repeating structural units (monomers) connected by chemical bonds. The polymers offer advantages such as flexibility, processability, and can be designed to have specific functions. The key difference is the composition that is used which does not contain any metal. In general, photocatalysts are inorganic metal oxides, such as TiO_2_ and metal sulfides.^93,94^ By contrast, metal-free polymer photocatalysts are made from 100% organic constituents without relying on metals. The range of wavelengths absorbed by the polymer and the amount of energy absorbed can be controlled by choosing a combination of monomers and their linkage in such a way that it will be suitable for certain applications and light sources, such as visible light. Metal-free polymer photocatalysts are a wide range of materials, all made from an organic polymer (which means the materials are composed of C, H, N, O, S elements, but contain no metal centers).^97^ These include conducting polymers (such as PANI and PPy discussed in Section 4.1), conjugated polymers and g-C_3_N_4_. Taking g-C_3_N_4_ and related porous organic frameworks as the metal-free part in polymer-based heterojunctions, their suitable band gaps and stability for photocatalytic applications are elaborated in the following.^98^

One of the major benefits of polymeric photocatalysts is that the organic building blocks of their polymers, such as C, N, O, and S, are more abundant and cheaper than the noble or heavy metals that are commonly used in traditional inorganic semiconductors. The removal of toxic heavy metals results in basically biocompatible and environmentally friendly catalytic matrices. Moreover, the pathways of organic synthesis are highly advanced, providing a large degree of freedom in modifying structure and functionalization to produce precisely defined surface areas, pore size distributions, and very accessible catalytic active sites. This is because many of these metal-free networks have outstanding chemical and long-term thermal stability in aggressive operating conditions from a thermodynamic point of view. In addition to pollutant degradation, these porous frameworks are increasingly used to address the climate crisis caused by the ever-increasing human CO_2_ emissions, known as anthropogenic CO_2_ emissions. After the solar excitation, the spatial separation of the photogenerated excitons leads to the anodic water oxidation to evolve oxygen, with the strongly oxidizing holes remaining in the valence band and the strongly reducing electrons promoted to the conduction band migrating to active surface centers, thus driving the cathodic conversion pathways of CO_2_ reduction. This electron transfer is energy-assisted and coupled reaction process can split water and fix carbon to produce valuable fuels, in addition to other products, from sunlight as shown in a schematic illustration in Fig. 6.

**Fig. 6:**
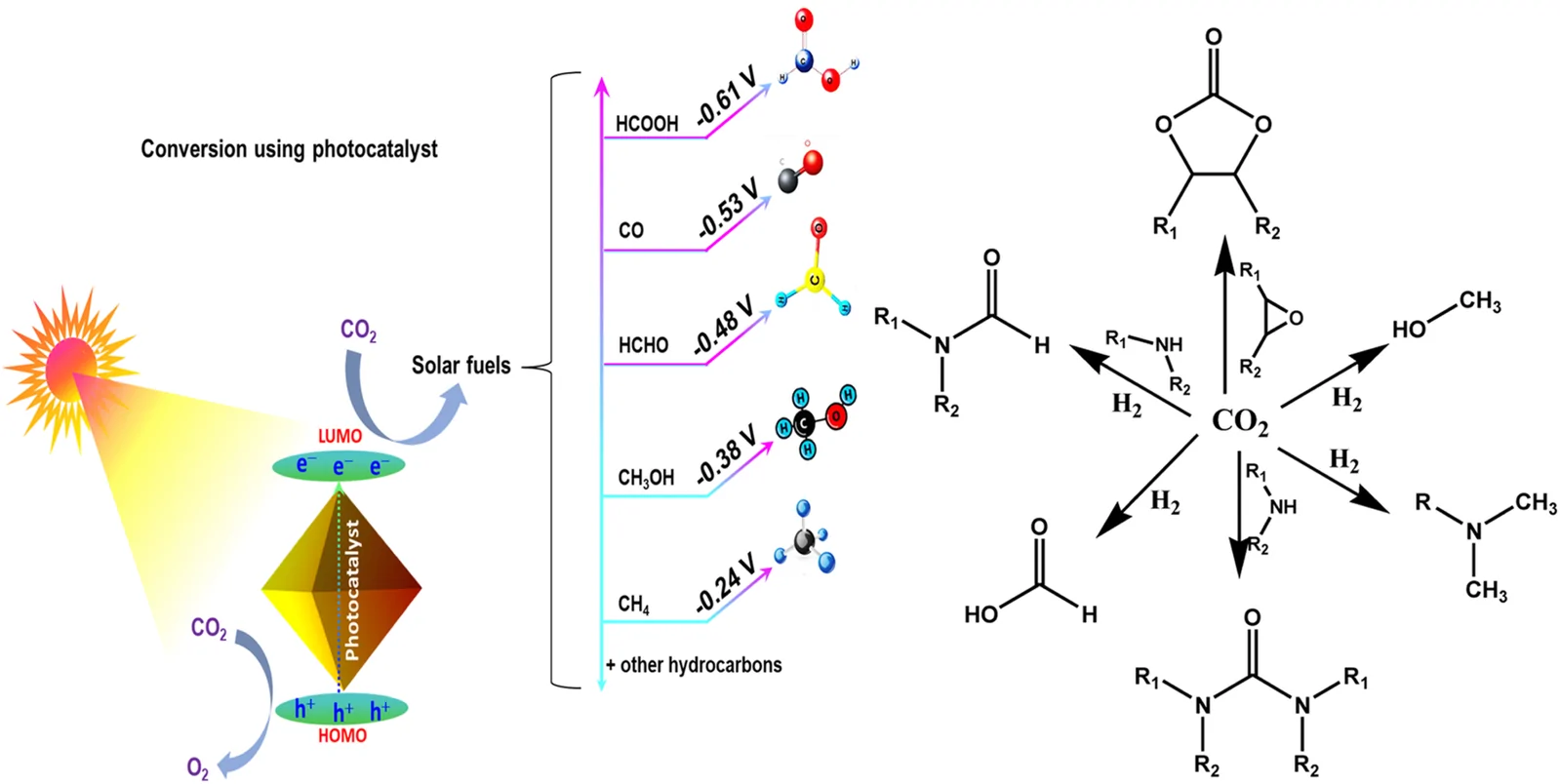
Schematic illustration of carbon dioxide photoreduction to solar fuels over polymer-based photocatalysts under solar irradiation. Adapted with permission from ref. 14*Nanomaterials* 2024, copyright 2024 under the Creative Commons attribution license.

A covalent organic polymer (COP) containing Mo has been used for reducing CO_2_ to CO and to CH_4_ at rates of 6.19 µmol g^−1^ h^−1^ and 1.08 µmol g^−1^ h^−1^, respectively.^99^ A polymer/inorganic hybrid In_2.7_S_4_ has been embedded in a POP to obtain ethylene alcohol with 67% conversion selectivity.^100^ With AuNP-decorated, Si-rich thiacalixarene-based POPs (a polymer/inorganic hybrid), a conversion of >4 µmol g^−1^ h^−1^ has been reached in a reaction time of over 4 hours.^101^ A CO_2_ to CO reducing capacity of CO-MOF-525 appeared 200.6 µmol g^−1^ h^−1^, and the CO_2_ to CH_4_ reducing capacity has been found ∼37 µmol g^−1^ h^−1^.^102^ These examples point to a competitive CO_2_-reduction activity which can be obtained by using a polymer in combination with a metal-containing component, rather than by the polymer used alone.^103^ The applications of metal-free polymer photocatalysts are diverse and growing, including environmental remediation, energy conversion, organic synthesis, photocatalytic polymerization, and water purification.

### 4.4 Emerging classes

In addition to well-known conducting, conjugated, and metal-free polymer photocatalysts, there are several new polymer classes that have recently gained interest as potential photocatalysts because of their special structure and improved photocatalytic activity. Polymer nanosheets have the following important merits compared to bulk polymers: a higher fraction of exposed active sites, longer lifetime of charge carriers, and shorter distance for transport of efficient charge migration. The single-component polymer nanosheets are cross-linked together in a manner that is highly cross linked, leading to excellent thermal and chemical resistance. For example, D–π–A conjugated microporous polymer (CMP) nanosheets with a thickness of ∼100 nm has been reported to show a HER rate of 0.21 mmol h^−1^ upon visible light irradiation with a catalyst amount of 25 mg.^84^ Likewise, the doped g-C_3_N_4_ nanosheets display enhanced photocatalytic activity, with a hydrogen production rate up to ∼9 mmol g^−1^ h^−1^ in the visible range.^85^ The important role of nanosheet morphology for improving photocatalytic efficiency has also been demonstrated by CTF nanosheets with amide functional groups, which exhibit much better oxygen evolution performance than their bulk counterparts.^104^

Another potential strategy for the development of highly efficient photocatalysts is the use of supramolecular assemblies. It is a strategy to spontaneously self-organize molecular building blocks into ordered architectures with hierarchical structures and non-covalent interactions such as hydrogen bonding, π–π stacking, electrostatic interactions, *etc.* Supramolecular assembly is efficient in the enhancement of the photocatalytic activity of carbon nitride materials in removing pollutants like Rhodamine B (RhB) through halogen substitution.^105^ One interesting example is the preparation of hydrogen evolution materials that are in the form of loofah-like carbon nitrides (LCN), which can undergo hydrogen evolution with a high rate of 4812 µmol h^−1^ g^−1^ and a high conversion efficiency of ∼97% when a platinum (Pt) co-catalyst has been used.^106^

As a result of their renewability, biodegradability, and environmental compatibility, bio-derived polymers have become sustainable alternatives. Natural polysaccharides like chitosan and cellulose are readily available renewables with extraordinary potential for photocatalytic purposes.^88^ The introduction of these bio-derivable polymers with outstanding stability thanks to their polysaccharide backbone and the absence of environmental impact, makes them particularly interesting for environmental remediation applications. The use of bio-templates and dopant sources has also been explored to enhance both the yield and performance, and up to 90% efficiency for carbon-doped g-C_3_N_4_ and C–N co-doped g-C_3_N_4_ materials has been achieved through the solvent-free route synthesis.^107^ Smart photocatalytic materials are the cutting edge of the field of self-healing and stimuli-responsive polymers. Self-healing polymers can repair their damage during photocatalytic reactions, which prolongs their operational lifetime and keeps them performing over a long time.^130^ Stimuli-responsive polymers tend to show adaptive behavior when external stimulus like pH, temperature, or light is applied to them, which creates tunable photocatalytic activity with multi-dimensional applications.^131^ An overview of the new classes discussed in this chapter is given in Fig. 7, and all of these indicate the growing range of polymer-based photocatalysis and the potential for developing new materials with custom-made functionalities for environmental and energy applications.^108^

**Fig. 7:**
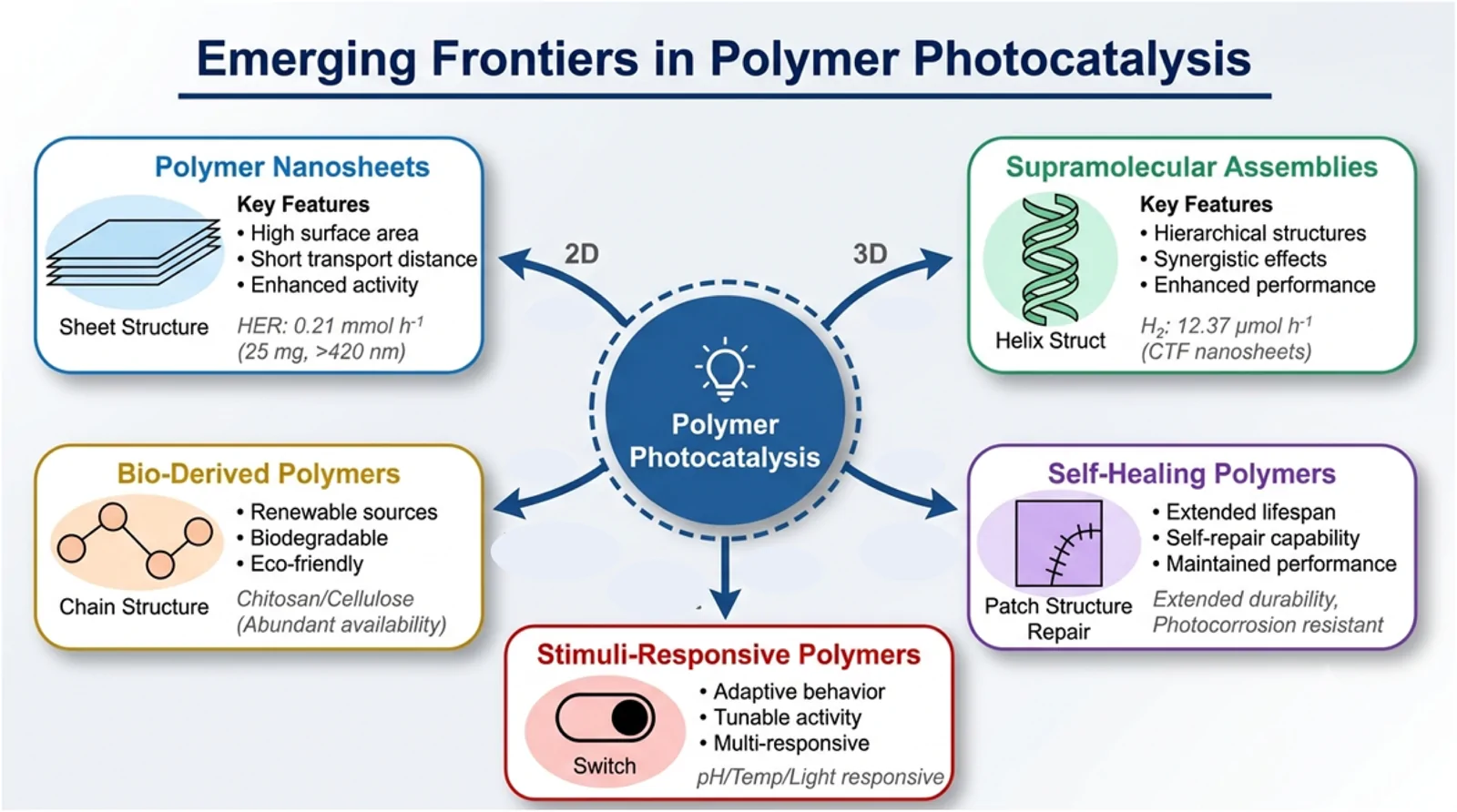
Schematic representation of the simultaneous photocatalytic water splitting and CO_2_ fixation pathways over a conjugated polymer matrix under solar irradiance.

## 5 Structure–property–performance relationships

The interface architecture, charge dynamics, and material properties all play a role in the photocatalytic performance of polymer-based heterojunctions. Clear structure–property–performance relationships are crucial for rational design, not development by trial and error.

### 5.1 Interface engineering and charge dynamics

The charge separation and transfer efficiency are directly controlled by interface engineering. The Type-II, Z-scheme and S-scheme mechanisms all depend on the correct alignment of the energy bands at the interface of the heterojunction. The thermodynamic feasibility as indicated by band alignment does not always imply the actual charge transfer pathway, however. The mechanism can only be confirmed by direct evidence obtained from techniques like transient absorption spectroscopy, *in situ* XPS, and Kelvin Probe Force Microscopy.^14^ Literature indicates that the use of only the quenching of photoluminescence or radical trapping for assignments is sometimes misinterpreted, as these methods may yield non-specific results.^14^ At least three complementary techniques of different types should be used for mechanistic assignment. These include determination of the band structure, photophysical studies, radical detection, and *in situ* characterization.^14,22^

As discussed in Section 2.2, the most important parameter that controls the charge carrier dynamics and photocatalytic activity of the exciton binding energy. It value for the representative classes appears in range ∼0.2–0.5 eV which allows efficient dissociation, higher binding energy polymers like PANI (∼0.5 eV) require heterojunction creation to achieve comparable activity.^14^

### 5.2 Material properties and performance

The efficiency of the heterogeneous photocatalytic system not only depends on the intrinsic electronic structure of the catalytic materials, but is also determined by the synergistic effect of the extrinsic operational parameters and the boundary reaction conditions. Optimization of these multi-variable factors is very important to maximize the use of photons and surface reaction kinetics.

#### 5.2.1 Extrinsic operational thresholds and reaction dynamics

The spectral profile and the number of photons available from the light source determine the rate of exciton generation and are called Extrinsic Operational Thresholds and Reaction Dynamics (EOTRD). From a thermodynamic perspective, the energy of the photons received by the semiconductor matrix should be more or equal to the optical bandgap of the semiconductor matrix, to be able to generate the electronic transition between the ground and excited states. New architectures like Au/TiO_2_ heterojunctions show increased photoresponse in the visible-light range of the spectrum, allowing photocatalytic water splitting based on surface plasmon resonance and transition energy changes in the absorption range.^106^ In addition, the number of electron–hole pairs generated increases linearly with the increase in light intensity (photon flux). This direct relationship, however, changes with high fluxes when the local accumulation of carriers increases the secondary radiative recombination processes and brings the quantum efficiency to a plateau and the reaction rate to a maximum.

In the photocatalytic pathways, the reaction mainly depends on photons, and can easily occur at ambient temperature (∼298 K), however, the underlying thermodynamics of the system is influenced by thermal fluctuations. Temperature controls the adsorption–desorption equilibrium of substrates on the catalyst surface. It also changes the liquid medium viscosity and affects the diffusion rate of reactive species. On the other hand, too high a thermal regime can cause deleterious phase changes, too rapid desorption of required reactants, or too rapid carrier trapping, which can result in early catalyst deactivation. The reaction parameters of pH, which can be changed in the aqueous matrix, plays a key role in both surface chemistry of the photocatalyst and the speciation of the target pollutants. Changes in pH away from the catalyst's point of zero charge (PZC) will change the surface charge density through protonation or deprotonation and so help to control the electrostatic attraction or repulsion of ionic substrates. In parallel, the solution pH affects the redox potentials of the chemical boundaries, affects the ionization states of organic pollutants, and affects the concentration of hydroxyl ions (OH^−^) that are key to forming highly ROS like hydroxyl radicals (˙OH).^107^ The rate of the reaction is dependent on the concentration of the photocatalyst. Concentrations can be raised above this to reduce light penetration and turbidity to reduce the efficiency. High concentrations may also lead to agglomeration of nanoparticles and thus to a decrease in surface area. A decrease in the agglomeration improves light adsorption.^108^ Moreover, the concentration of reactants should be taken into account. At high concentrations, however, the number of active sites on the catalyst surface may become saturated, and the rate of the reaction will not increase further. Monitoring of the presence of electron acceptors/donors should be carried out through co-catalysts. Co-catalysts such as noble metals (Pt, Au, *etc.*) or other semiconductors can also increase charge separation by trapping the electrons or holes, by facilitating charge transfer at the interfaces and by increasing the overall efficiency.^109^

The importance of dissolution of oxygen lies in the fact that oxygen is an essential electron scavenger, which not only helps to prevent electron–hole recombination but also helps to produce ROS such as superoxide radical.^110^ Reaction time is extremely crucial. It is important to get enough irradiation time to get the desired conversion or degradation. It is important to know and optimize these parameters to design efficient photocatalytic systems for different applications. When studying photocatalysis, researchers tend to try to fine-tune the properties of the photocatalyst and precisely control the reaction conditions to get the best possible results.

#### 5.2.2 Intrinsic threshold indicators affecting photocatalytic performance

There are various factors affecting the overall performance of polymer-based photocatalysts. A three-dimensional repeating ordered pattern is called crystallinity. It improves the mobility for carriers by enhancing the π-conjugation. Waste of energy during recombination is suppressed by the efficient transport to the active sites of the surface. The accessible surface area is known as porosity. More surface area is exposed by the factor of porosity. The interaction of the molecules with other substances is defined by the chemical functionality. It is the basic parameter to manage the charge transfer, band gaps, binding energy, *etc.* A green photocatalyst Mpg-C_3_N_4_ is obtained with good crystallinity.^109^ Additionally, with controlled other variables, 7.3 times more catalytic activity is observed at a strong wettable porosity with the presence of ascorbic acid and photo deposited Pt.^110^ One important parameter which affects photocatalytic activity is the surface area and also the number of accessible active sites for the surface reaction and reactant adsorption.^111^ Specific surface area (SSA) is generally expected to be higher for better photocatalytic performance because of the increased number of reaction sites and efficiencies of light harvesting due to the multiple scattering effect. Surface area/activity is not linear, however. Mesoporous structures (2–50 nm pores) generally possess the lowest diffusion resistance and the highest surface area, while microporous structures (<2 nm pores) could have micropores, which would decrease the accessibility of active sites due to diffusion limitation.^112^ In addition, surface area does not correlate with activity; defects and crystallinity of the surface, as well as pore connectivity, have very important roles to play.^113^ Studies that show increases in activity only because of an increase in surface area should be approached with caution because other morphologic and structural changes are often associated with changes in surface area. This optimum surface area varies with the nature of the reaction, the composition and nature of the catalyst and the operating conditions.^114^

The redox thermodynamics governing the placement of the LUMO/HOMO are detailed in Section 2 whereas the explanation also involves the case of hydrogen evolving systems for which there is an additional redox couple, the H^+^/H_2_ (0 V *vs.* NHE). Recombination that depends on the binding energy of an exciton, as well as on mobility of the carriers, can be addressed at the material level, with regards to the formation of heterojunctions (Type-II, Z-scheme, S-scheme), deposition of co-catalysts, and defect engineering.^120^ The solar energy conversion technologies, such as photovoltaics and photoelectrolysis, performance is improved by addressing methodologies to tackle this gap. If they absorb the NIR or visible light of the solar spectrum, they could create the heterojunctions for efficient operation.^121^ The excitation binding energy and e/hole pair recombination rate are the key factors that determine the charge kinetics. The more the charge-carrying kinetics, the faster the surface redox reaction occurs. Resistant to photocorrosion (photo stability) is the primary determinant; it may lead to the decomposition of the material itself. So, for durability, strategies should be introduced for a longer span.

The relationship between extrinsic reaction conditions and intrinsic material properties governs the overall photocatalytic efficiency. While extrinsic factors can be optimized for specific applications, intrinsic properties require careful synthesis strategies and material design. A comprehensive summary of the key extrinsic and intrinsic factors affecting photocatalytic performance, along with their effects and typical ranges is provided in Table 5. These parameters collectively decide the overall photocatalytic efficiency and must be carefully optimized for specific applications. For large-scale applications, development can be accelerated by commanding the correlation for charge transfer pathways and morphology for polymers for planet-friendly solar-energy conversion applications. The reaction conditions should be taken into account for an effective process to occur.

**Table 5:** Key parameters that affect the performance of polymer-based photocatalysts

Factor	Category	Optimal condition	Typical range	Effect on performance	Reference
Light wavelength	Extrinsic	*λ* ≤ 1240/*E*_g_ (nm)	UV (200–400 nm), visible (400–700 nm)	Determines e^−^/h^+^ generation	115
Light intensity	Extrinsic	Moderate intensity	0.1–5 Sun	Increases e^−^/h^+^ generation	115
Crystallinity	Intrinsic	High crystallinity	Amorphous to highly crystalline	Improves carrier mobility	116
pH	Extrinsic	Pollutant-dependent	0–14	Affects surface charge	117
Temperature	Extrinsic	Room temp. to moderate	20–80 °C	Affects adsorption kinetics	115
Co-catalysts	Extrinsic	Optimized loading	0.1–5 wt% (Pt, Au, *etc.*)	Enhances charge separation	118
Dissolved oxygen	Extrinsic	O_2_ saturated	Air/O_2_ saturated	Prevents e^−^/h^+^ recombination	115
Photostability	Intrinsic	High stability	Photocorrosion resistance	Determines catalyst durability	117
Porosity	Intrinsic	Mesoporous (2–50 nm)	Microporous (<2 nm)/mesoporous (2–50 nm)	Increases accessible surface area	116
Band gap	Intrinsic	Narrower for visible light	1.5–3.2 eV	Determines the light absorption range	119
Charge carrier kinetics	Intrinsic	Fast separation, slow recombination	ps–µs lifetimes	Determines surface reaction rate	119

### 5.3 Methodological consideration for performance reporting

Direct comparison of the photocatalytic performance from literature is complicated by several factors. Different types are available (UV lamps, Xenon lamps, LEDs, solar simulators) and have different spectral distributions, intensities, and irradiation geometries.^122^ The amounts of catalyst used, the concentration of pollutants, temperature, pH, and reaction times vary widely among the studies—sometimes by orders of magnitude. As a result of this, it is impossible to compare reported performance, such as degradation percentages, hydrogen evolution rates, and quantum yields, from study to study without explicitly re-matching the conditions in the experiments. This is a basic constraint of the field in terms of being able to create a reliable structure–property–performance relationship.^77^

While the sacrificial electron donors (TEOA, methanol, ascorbic acid) are important for hydrogen evolution, they cannot be considered as overall water splitting.^123^ High reported percentages in degradation studies may be due to the disappearance of the parent compound, not necessarily complete mineralisation; the transformation products could be more toxic than the parent compound.^124^ The number of cycles of 3–5 is not a sufficient number to guarantee long-term operational stability for recyclability. Reporting a process in the field would benefit from having standardised procedures that define all critical experimental conditions and important performance metrics.^77^

## 6 Technological applications and scope

The tunability of their molecular structures, their inherent ability to be soluble and to harvest light efficiently, make polymer photocatalysts promising. These materials can be rationally engineered to target a specific pollutant and reaction conditions and applied in three main areas: pollutant removal, energy conversion, and/or organic synthesis, as shown in Fig. 8.

**Fig. 8:**
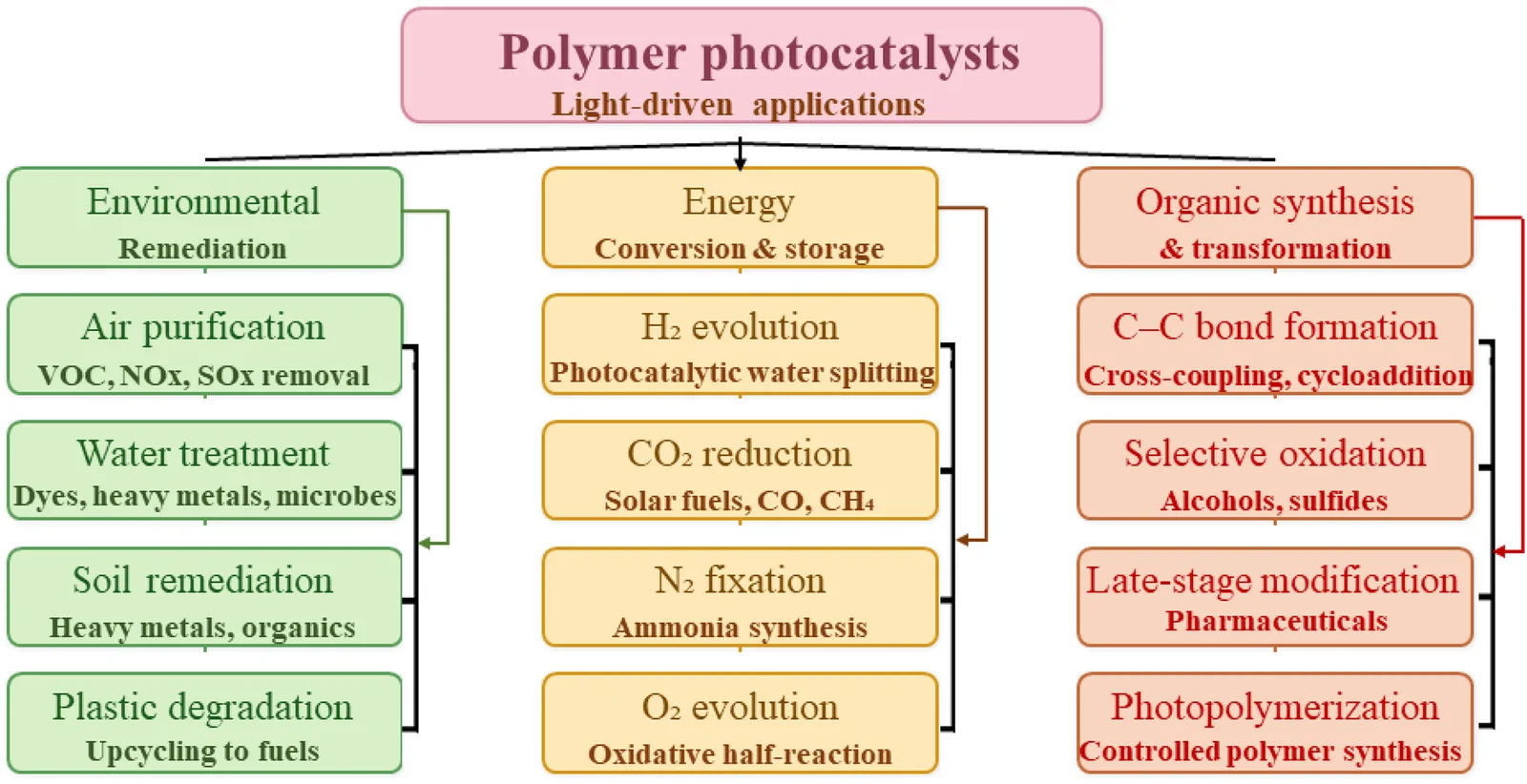
Overview of light-driven applications of polymer-based photocatalysts in environmental and energy sectors, along with organic transformation and chemical synthesis.

### 6.1 Environmental remediation and disinfection

Polymeric carbon nitride (PCN) surfaces have been proven to have outstanding performance in the destruction of atmospheric and aquatic pollutants by oxidation. For example, ultra-small SnO_2_ quantum dots attached to PCN/SnO_2_ have been used to convert harmful gaseous nitric oxide (NO) to harmless nitrate in the presence of simulated solar light with 68% efficiency.^98^ Likewise, potassium doped matrices (K/PCN) showed a 95% efficiency of degradation of volatile formaldehyde under visible-light illumination.^103^ In addition to VOCs, mesoporous PCN architectures were also able to catalyze the deep desulfurization of fuel models, with a 100% conversion of dibenzothiophene (DBT) to dibenzothiophene dioxide (DBTO_2_).^100^ Although these encouraging reference data exist, a major challenge is the poor record of operational stability of these PCN-based systems when they are operated under continuous flow conditions, as well as the lack of systematic study of the competitive inhibitory effects of atmospheric humidity on active surface sites.^98–102^ Narrow-bandgap materials have often been used to realize these polymeric configurations that increase the light-harvesting window. Spinel ferrite materials like zinc ferrite ZnFe_2_O_4_ have a well optimized narrow optical bandgap of 1.4 eV, which is able to absorb all the visible light in the solar spectrum. But intrinsic limitation of pure ZnFe_2_O_4_ is the fast charge-carrier recombination rate, which is thermodynamically favored, and is generally the problem that leads to fewer potential applications in advanced wastewater treatment systems and water purification based on quantum efficiencies.^103^ Phenolics are highly toxic aromatic contaminants and are still one of the most important measures to assess these AOP technologies. The combination of TiO_2_ with exfoliated reduced graphene oxide (ERGO) in composite matrices resulted in a 73% degradation efficiency after photo-excitation under UV light, while after visible-light illumination, the degradation efficiency reached 90% due to the shift in the absorption edge to the red and the decrease in the threshold bandgap of the carbonaceous support.^104^ In addition, these systems are highly efficient at reductively immobilizing heavy metal ions. The optimal dose of the TiO_2_ catalyst was found to be ∼1g L^−1^ and under the optimized conditions (pH 11), 88% of aqueous cadmium ions (Cd^2+^) were removed after 1 hour of exposure to the UV source. Lead ions (Pb^2+^) were also effectively captured with 94% efficiency, in the same hydrodynamic and radiative conditions, thanks to the simultaneous cooperative action of reduction processes mediated by electrons, and surface adsorption processes.^105^ It also involves the degradation of soil contaminants. Similar to water remediation, polymer photocatalysts can be employed to break down organic pollutants present in contaminated soil. This can be achieved through slurry reactors or by direct application to the soil surface, followed by light irradiation. Heavy metals are naturally present in the Earth's crust, but some lands are enriched with these metals. Physical methods can rapidly remove heavy-metal pollutants. Separating As and Hg requires temperatures of ∼1000–2000 °C, which extracts hazardous material from contaminated soil. Temporary effectiveness is provided by the chemical methods, which are economical. The incorporation of heavy metals into plants can be minimized by various chemical methods. The synergistic effect is produced by the combination of physical and chemical methods. Soil washing is the basic technique; it is a highly efficient process as it involves the precipitation of heavy metals. Volatilization, phytostabilization, and rhizofiltration are the biological methods, which are beneficial for the limited area with limited pollutant concentration of heavy metals.^125^

Photocatalytic degradation of plastics breaks down plastic polymers into smaller, less harmful molecules or even valuable monomers for recycling. Semiconductors like TiO_2_ and ZnO incorporated into polymer matrices can produce ROS that degrade plastic chains. Upcycling of plastic waste, which involves converting plastic waste into value-added chemicals or fuels using photocatalytic processes. For instance, LDPE has been photocatalytically converted into liquid fuels using Ni–TiO_2_–Al_2_O_3_ catalysts.^126^ In addition to heavy metals, microorganisms and antibiotics also contribute to environmental safety concerns. TiO_2_ nanoparticles are incorporated with Si, and multifunctional synthetic membranes, such as PVDF-HFP, are used to create a safe environment from microbes. The growth of both Gram-positive and Gram-negative bacteria, as well as their impact on the environment, is hindered by this material. The efficiency of Ag/TiO_2_/5-aminosalicylic acid reduces the growth of *E. coli* and *S. epidermidis* by a factor of 2 log_10_ and 0.5 log_10_, respectively.^127^ ROS are produced by the interaction of light with specific materials such as semiconductors. These products effectively destroy bacteria and microbes.^128^ Photocatalytic performance parameters evaluated for environmental remediation applications with polymer-based photocatalytic systems are given in Summary Table 6.

**Table 6:** Photocatalytic degradation of organic pollutants in aqueous solution

Application	Pollutant target	Catalyst loading (mg L^−1^)	Photocatalyst system	Light source	Pollutant conc. (mg L^−1^)	Efficiency	Wavelength/filter	Reaction time	Ref.
Water treatment	RhB	200	PANI/BiOBr-0.2	>420 nm	10	99.9%	>420 nm	75 min	129
Water treatment	MO	100	PPy/TiO_2_	500 W Xe	20	100%	500 W	60 min	82
Water treatment	Malachite green	100	BiOBr-Ag-PPy	150 W halogen	10	97%	150 W	120 min	83
Water treatment	Phenol	100	PPy/Bi_2_WO_6_	500 W Xe	10	100%	500 W	120 min	84
Water treatment	Ciprofloxacin	200	PANI/Bi_12_O_17_Cl_2_	300 W Xe	10	91%	300 W	120 min	130
Water treatment	RhB	200	PANI/Bi_12_O_17_Cl_2_	300 W Xe	10	99%	300 W	100 min	130
Water treatment	MO	100	PANI/BiOCl	Visible	20	67%	>420 nm	20 min	131
Water treatment	RhB	100	ZnO-microrods/PPy	Visible	10	—	>400 nm	—	85

Beyond the aqueous pollutant degradation, polymer photocatalysts have also been reported for air purification demonstrating 68–95% NO_*x*_ and formaldehyde removal under solar or LED illumination,^132–137^ heavy-metal removal with 88% Cd^2+^ and 94% Pb^2+^ removal under UV, bacterial disinfection exhibiting 1.5–2 log_10_ reduction under visible light, and plastic upcycling.^126,127,138^ These applications are qualitatively discussed below because their experimental conditions differ substantially from those in Table 6 and do not permit direct quantitative comparison.

It is important to note that the degradation efficiencies provided in this section and in Table 6 are from different experimental conditions. The variation of catalyst loading, pollutant concentration, light intensity and wavelength, reaction time, pH and reactor geometry among various studies are very significant. The absolute degradation percentage is not always the most suitable parameter for making direct comparisons between different systems without consideration of these parameters.^139^ It would be useful to have reportable protocol(s) to the field that outline all important experimental variables. In addition, high degradation percentages usually mean disappearance of the parent compound, not true remediation – best to use TOC analysis to confirm true remediation.^140^

#### 6.1.1 Critical assessment of remediation completeness

A fundamental distinction must be made between the reduction of the concentration of primary pollutants, which is generally measured through UV-vis absorbance decay, and complete mineralization into non-toxic inorganic end products, such as CO_2_, H_2_O, NO_3_^−^, and Cl^−^, to undertake a rigorous evaluation of photocatalytic performance in environmental remediation.^141,142^ This distinction is summarized in Fig. 9. Degradation is the loss of the parent compound only whereas remediation also includes evidence of mineralization, identification of transformation products, and the acceptable ecotoxicity.^143,144^

**Fig. 9:**
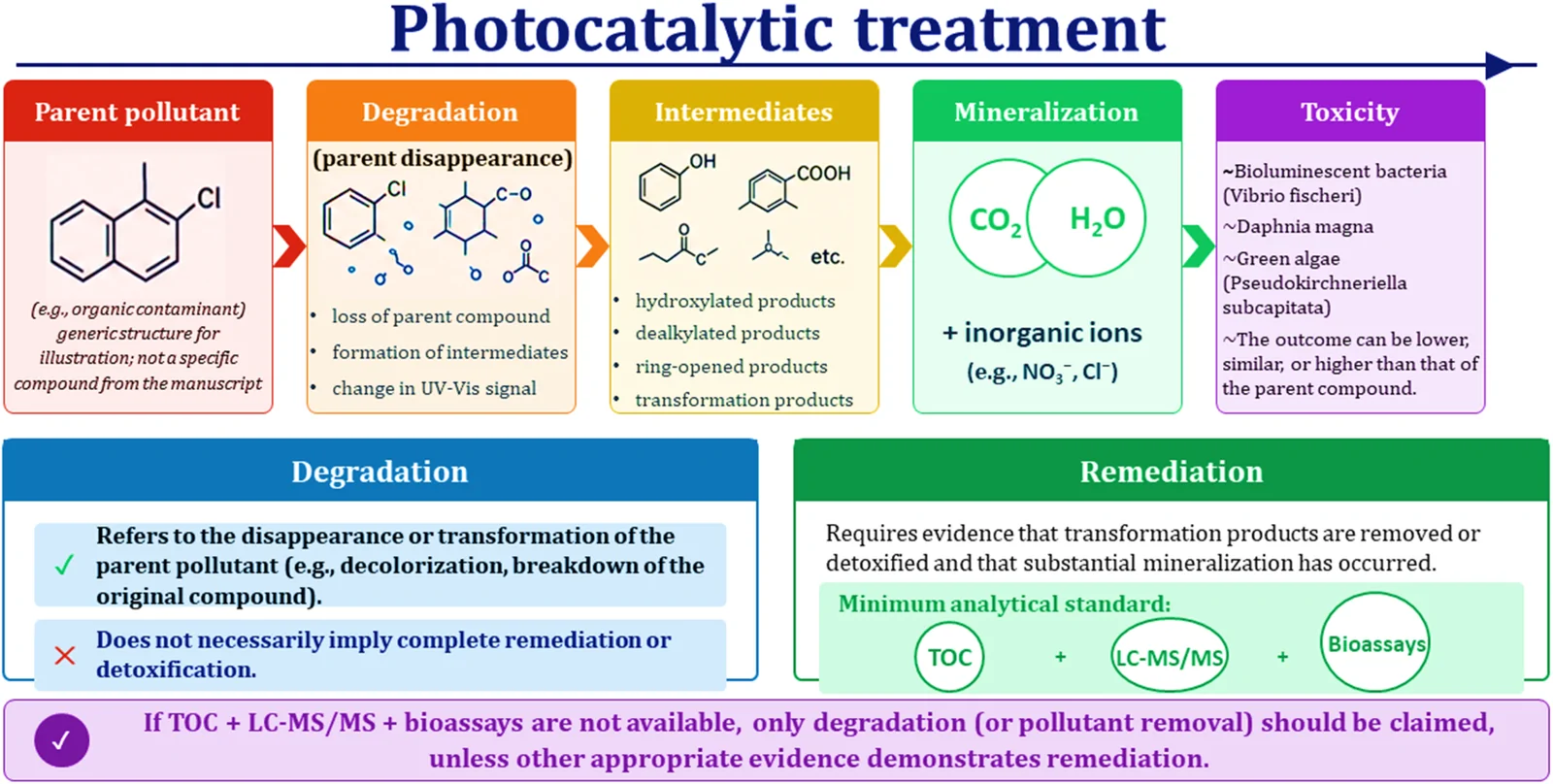
Photocatalytic treatment pathway from parent pollutant to degradation, intermediates, mineralization, and toxicity. Degradation refers to parent pollutant disappearance (UV-vis/HPLC), while remediation requires TOC, LC-MS/MS, and bioassays together. Without all three, results should be reported as degradation only.

Literature reports indicate that parent-compound disappearance does not necessarily imply full remediation, since photocatalytic research has been normally focused on the degradation of the parent-compound data, and identification and quantification of byproduct species are not always carried out.^141,143,145^ Transformation products may be formed in environmental matrices, react with reactive species along with the parent contaminant, have different apparent degradation rates and may even be more toxic than the contaminant.^142,146^ One drawback of the literature on the use of polymer-based photocatalysts is that primary decoloration or parent molecule disappearance (concentration ratio, *C*/*C*_0_) is used to represent overall remediation efficiency. Decolorization or a low *C*/*C*_0_ value, especially when measured only by UV-vis, can overestimate remediation success. Early cleavage of chromophores suppresses absorbance without breaking down the carbon skeleton. Dissolved intermediates remain in solution and continue contributing to organic carbon.^147–149^ This preliminary cleavage leads to fast disappearance of the specific light absorbance at *λ*_max_, which gives the impression of a complete removal of the pollutant in a short reaction time. High-performance liquid chromatography-tandem mass spectrometry (LC-MS/MS), however, shows that the parent pollutant is only transformed into a mixture of reaction intermediates, such as hydroxyaromatics, quinones and ring-opened carboxylic acids and short-chain aliphatic aldehydes, which are still soluble in the treated effluent.^141,142^

Recent mechanistic studies demonstrate the need for intermediate tracking. In addition to direct one-step mineralization, complex product mixtures have been identified during photocatalytic treatment by Liquid Chromatography-High Resolution Mass Spectrometry (LC-HRMS), such as hydroxylated products, ring-opened products, dealkylated products, oxidized products, and defluorinated products.^148–150^ For instance, thirteen transformation products were identified in the degradation of ciprofloxacin before mineralization was confirmed (by TOC and ion release analysis).^151^ In the case of ceftazidime, nineteen transformation products were identified and TOC values revealed that the photocatalysis treatment resulted in greater mineralization than other treatments studied.^148^ A difference between parent removal and mineralization can also be seen in the reactor study. Within the continuous-flow photocatalytic reactor, degradation of parent-compounds was always higher than the removal of TOC, which showed that there was no significant decrease in the concentration of transformation products even with the significant decrease in the concentration of the target pollutant.^147^

The photocatalytic degradation of azo dyes leads to the breaking of the azo bond to give the colorless aromatic amines, which are more toxic and carcinogenic than the parent dye molecules and cannot be detected with the usual UV-vis spectrophotometric method. Likewise, a photocatalytic process to break the azo (–N=N–) or the anthraquinone chromophore yields aromatic amines and quinone intermediates which can, temporarily, be found in treated effluents.^144^ This is especially alarming as the aromatic amines are more toxic and carcinogenic than the dye molecules themselves. The toxicity needs to be measured but not assumed because the photodegradation pathway, in addition to the operating conditions, can either increase or decrease the toxicity of the mixture, or even create a mixture that is as or more toxic than the original compound.^143,144,150^ Sulfamethoxazole was more toxic than the original compound in bioassays following photodegradation in each of the tested photodegradation systems, even after contaminant removal.^143^ In contrast, the degradation of metronidazole using ZnO/PANI and TiO_2_/PANI showed a decrease in the toxicity of the degradation products, but the degradation products were still found after complete degradation, which means that the degradation of metronidazole was not complete and that intermediates were still present.^144^ The luminescent marine bacterium *Vibrio fischeri*, a freshwater microcrustacean (*Daphnia magna*), or a green microcrustacean (*Pseudokirchneriella subcapitata*) are sensitive bioassays of effluent safety.^143,147,149^ The degree of toxicity of the transformation products produced in the photocatalysis process is one of the key factors in accepting the process for wider applications.

This is a significant difference between primary elimination and total remediation, which also applies to atmospheric applications. Primary NO conversion rates under visible light for gas-phase photocatalytic air purification may be deceiving if the product distribution is not carefully quantified. Partial photo-oxidation of NO gives rise to nitrogen dioxide (NO_2_) which is a highly toxic gas and has a lower threshold limit value than NO, while complete mineralization is possible by selective 4-electron oxidation to nitrate (NO_3_^−^).^152^ Therefore, future research that compares the efficiency of polymer photocatalysts for environmental applications needs to move beyond reporting on isolated *C*/*C*_0_ to a dual-metric approach that includes high-resolution mass spectrometry for intermediate identification, TOC and chemical oxygen demand (COD) for mineralization assessment, and standardized bioassays for ecotoxicity verification.^141,143,145,151,153^ The photocatalytic community needs to develop beyond reporting degradation percentages to a complete remediation assessment, which involves the identification of intermediates and toxicity assessment as well as mineralization.^152^

### 6.2 Photocatalytic hydrogen evolution and overall water splitting

The HER reported in this section and summarized in Table 7, were measured in the presence of a sacrificial electron donor (usually H^+^), in addition to a co-catalyst (usually Pt), and refer to the hydrogen evolution half-reaction, not overall water splitting. In the field, sacrificial-donor systems are commonly employed to isolate and measure the reductive half-reaction because the sacrificial electron donor is oxidized (preferentially and irreversibly) and is not able to undergo electron–hole recombination or back-reaction. Recent publications have challenged the feasibility of continued use of sacrificial agents for scalable production of hydrogen by photocatalytic water splitting, however, fearing that continued use of sacrificial agents would be a diversion of resources and reduce confidence in photocatalytic hydrogen production as a scalable climate solution.^154^ The rates reported are not directly comparable to, nor equivalent to, a real overall water splitting system which produces H_2_ and O_2_ in the correct 2 : 1 ratio without the consumption of a reagent. The source of the sacrificial donor and the co-catalyst used are indicated in Table 7. Water splitting systems that are shown to split water overall without a sacrificial agent are specifically identified as such.

**Table 7:** Performance of polymer photocatalysts for solar-to-chemical energy conversion

Energy pathway	Photocatalyst system	Sacrificial agent	Reaction conditions	Performance metric	Ref.
H_2_ evolution	Y_4_ (heptazine-CMP)	TEOA	300 W Xe lamp, pH ∼7	27 mmol g^−1^ h^−1^	157
H_2_ evolution	CYANO-COF	Ascorbic acid	300 W Xe lamp, *λ* > 420 nm	60.85 mmol g^−1^ h^−1^	95
H_2_ evolution	TSO_2_@TiO_2_	Methanol	300 W Xe lamp	11 220 µmol g^−1^ h^−1^	89
H_2_ evolution	T-CN-0.010	TEOA	300 W Xe lamp, *λ* > 420 nm	1.618 mmol g^−1^ h^−1^	90
H_2_ evolution	UCCN	TEOA	300 W Xe lamp, *λ* > 420 nm	9.7 mmol g^−1^ h^−1^	91
H_2_ evolution	CNSO-20	TEOA	300 W Xe lamp, *λ* > 420 nm	5.02 mmol g^−1^ h^−1^	92
H_2_ evolution	Tz-COF-3	TEOA	300 W Xe lamp, *λ* > 420 nm	43.2 mmol g^−1^ h^−1^	93
H_2_ evolution	BTH-3	TEOA	300 W Xe lamp, *λ* > 420 nm	15.1 mmol g^−1^ h^−1^	94
H_2_ evolution	D–π–A CMP nanosheets	TEOA	Visible light, 25 mg catalyst	0.21 mmol h^−1^	104
H_2_ evolution	Doped g-C_3_N_4_ nanosheets	TEOA	Visible light, Pt co-catalyst	∼9 mmol g^−1^ h^−1^	106
H_2_ evolution	TiO_2_@2PEDOT	Methanol	UV-vis light	1.37 mmol g^−1^ h^−1^	86
H_2_ evolution	TiO_2_@2PPy	Methanol	UV-vis light	2.09 mmol g^−1^ h^−1^	86
H_2_ evolution	TiO_2_@5PAn	Methanol	UV-vis light	3.1 mmol g^−1^ h^−1^	86
O_2_ evolution	Fe^2+^-PHI	AgNO_3_ (electron acceptor)	300 W Xe lamp, *λ* > 420 nm	11.2 µmol h^−1^	158
O_2_ evolution	10ZnS/SPI	No sacrificial agent	300 W Xe lamp	217 µmol g^−1^ h^−1^	96
CO_2_ reduction	Mo-COP	TEOA	300 W Xe lamp	CO: 6.19 µmol g^−1^ h^−1^, CH_4_: 1.08 µmol g^−1^ h^−1^	99
CO_2_ reduction	POP-In_2_._77_S_4_	TEOA	Visible light	C_2_H_5_OH: 67% selectivity	100
CO_2_ reduction	AuNP-POP	TEOA	Visible light	∼7 µmol g^−1^ h^−1^ (CO)	101
CO_2_ reduction	Co-MOF-525	TEOA	300 W Xe lamp	CO: 200.6 µmol g^−1^ h^−1^, CH_4_: ∼37 µmol g^−1^ h^−1^	102
CO_2_ reduction	LaYAgO_4_-G-TiO_2_	TEOA	Visible light	CH_3_OH: ∼13% conversion	103

The conversion of solar energy into chemical fuels represents the most transformative potential of polymer photocatalysts. Polymer-based photoanodes have demonstrated 20-fold higher photocurrent compared to conventional systems, enabling simultaneous wastewater treatment and electricity generation. Photocatalytic water splitting for hydrogen production in such a way that photocatalysis can be employed to split water into hydrogen and oxygen using sunlight and a semiconductor catalyst. This process, often referred to as artificial photosynthesis, offers a green and renewable route to produce hydrogen, a clean energy carrier. As metal-free photocatalysts, PCN is composed of monomers called amine-bridged heptazine molecules.^155^ In D–A structured compounds, heating above 500 °C stabilizes charge carriers and enables H_2_ evolution.^156^ Heptazine-based CMPs are a promising material. Y_1_ and g-C_3_N_4_ evolve less hydrogen, which is 57 and ∼1930 times less than Y_4_, respectively. The reported HER of Y_4_ is 27 mmol g^−1^ h^−1^.^157^ The oxygen evolution reaction is enhanced by the poly-heptazine imides. Moreover, the doping strategies also improve the O_2_ production by introducing metallic compounds. The Fe^2+^ incorporation with poly-heptazine imides evolves 11.2 µmol h^−1^.^158^ Yields of CO_2_-photoreduction products must be interpreted with caution. Reaction rates in this field are often low and the presence of carbon-containing products due to the carbon-containing components used in the catalyst synthesis, adhesives, sealants or from incomplete cleaning of the reactor can result in false-positive carbon products. To verify that CO, CH_4_, or alcohol products genuinely originate from CO_2_ reduction rather than an exogenous carbon source, isotope-labelling verification using ^13^CO_2_ is recommended along with dark and inert-gas (N_2_/Ar) blank controls.^154^ Where such verification is reported in the source publications listed in Table 7, it is noted; otherwise, the yield should be read as a literature-reported rather than fully authenticated CO_2_-reduction rate.

Photocatalysis can be used to convert carbon dioxide, a major greenhouse gas, into valuable fuels and chemicals. With the help of sunlight and a suitable photocatalyst, CO_2_ can be reduced to hydrocarbons such as methane, methanol, ethanol, and other useful products like formaldehyde (HCHO) and formic acid (HCOOH). This approach not only helps in mitigating CO_2_ emissions but also provides a pathway to produce fuels and chemicals sustainably. CO_2_ reduction sites are provided by the Cu_2_O/PCN. It yields ∼14 µmol g^−1^ of carbon, compared to the pure PCN.^159^ Another utilization of polymer-based heterojunction photoanodes involves their application in the field of photocatalytic fuel cells (PFCs) where the heterojunction functions as an anode for pollutant oxidation and a cathode employs for the generation of electricity. Besides achieving the high photocurrents derived from degradation of pollutants, heterojunction photoanodes can produce up to 20 times higher photocurrent in comparison to the single-component polymeric photoanodes.^160^

Solar fuel production is achieved in such a way that photocatalysis plays a crucial role in the broader concept of solar fuel production, which aims to convert sunlight into storable chemical fuels. Besides hydrogen and the reduction of CO_2_ to hydrocarbons, photocatalysis can be employed in other solar fuel production pathways, such as the production of ammonia from nitrogen and water. Nitrogen fixation is essential; it is initiated by the preparation of NH_3_. It is prepared at high temperature and pressure of about 400–500 °C and 150–250 atm, respectively. Except for nitrogen-based fertilizers, there is less consumption of N_2_. Therefore, the conversion of N_2_ to NH_3_ is necessary.^161^ Some photocatalytic materials can also exhibit up-conversion or down-conversion properties, allowing for better utilization of the solar spectrum by converting low-energy photons to higher energies or *vice versa*, which can be more efficiently absorbed by the solar cell. Photocatalysis offers a versatile platform for addressing energy challenges by enabling the conversion of solar energy into clean fuels, reducing greenhouse gas emissions, and even integrating with fuel cell technology for simultaneous energy generation and environmental remediation. The potential for these forms of energy applications is yet to be fully realized, and it is essential that research and development of photocatalytic materials and designs of reactors is continued.

### 6.3 Organic transformation and chemical synthesis

The photocatalytic transformation and synthesis of organic molecules has become a powerful and sustainable method. It utilizes light energy to catalyze chemical reactions, typically under mild conditions, with a number of benefits over conventional methods. The photocatalyst is the most important component of a photocatalytic process because it absorbs the light and catalyzes the reaction but itself is neither consumed in the reaction nor degraded by it. The photocatalyst gets excited when it absorbs photons of a certain wavelength. This excited state photocatalyst can then react with organic molecules in a number of ways. The exited photocatalyst can transfer one electron to the substrate or one electron from the substrate to create a radical ion that can be very reactive and undergo additional charge transfer reactions.

The evolution rate for H_2_ for all the catalysts in Table 7 has been measured with sacrificial electron donors (TEOA, methanol, ascorbic acid or as indicated). Values cannot be compared directly to general water splitting systems without sacrificial agents since the sacrificial donors lower the thermodynamic barrier and are used up in the reaction.^154^ AgNO_3_ is used here as a sacrificial electron acceptor for the evolution of O_2_. CO_2_ reduction products need to be established by isotope labelling experiments to ensure that reduction products detected are derived from CO_2_ and not from other sources, such as degradation of the catalysts or organic impurities.

Photocatalysis has been widely used for several organic transformations. As photocatalysis can be used to perform many cross-coupling reactions, cycloadditions and alkylation reactions, it can be used to form complex carbon skeletons, such as C–C bond formation.^118^ C-heteroatom bond formation, where reactions such as amination, etherification, and halogenation can be efficiently carried out using photocatalytic methods.^162^ Oxidation and reduction reactions, such as photocatalysis, enable the selective oxidation of alcohols, sulfides, and other functional groups, as well as the reduction of carbonyl compounds and nitro groups.^163^ Functional group transformations, such as isomerizations, rearrangements, and decarboxylations, can be achieved under photocatalytic conditions.^164^ Late-stage functionalization, as the mild conditions often associated with photocatalysis allow for the selective modification of complex molecules, including pharmaceuticals and natural products, at a late stage of synthesis. Polymerization as photocatalysis can be used to initiate and control polymerization reactions.^165^ The reported HER were generated using electron donors as sacrificial components as noted above (either TEOA, methanol, ascorbic acid or as noted). The rates are not to be equated with the overall rate of water splitting, as these are the sacrificial agents that are supplying the electrons, which are much more effective in reducing thermodynamic barriers.^166^ CO_2_ reduction reactions require carbon source verification using the isotope labeling method ^13^CO_2_ or any other labeled carbon source to ensure that the products detected are from the CO_2_ and not from the degradation of the catalyst and/or organic contaminants.^167^ The results of studies without such proof should be used with caution. As in environmental remediation, it is difficult to directly compare absolute values of performance because of the great differences shown in experimental conditions between studies.^168^

## 7 Challenges and strategies for the present time

Photocatalysis is a novel technology for environmental remediation and energy conversion, which exploits light energy for chemical reactions to semiconductor catalysts. While offering numerous advantages, the long-term stability and durability of photocatalytic processes face several challenges that hinder their widespread industrial applications. The photocatalyst, the core of the process, can lose its activity over time due to photocorrosion, surface fouling, poisoning, thermal degradation, agglomeration, sedimentation, and mechanical attrition.^7^ The major deactivation pathways and corresponding mitigation strategies are summarized in Fig. 10.

**Fig. 10:**
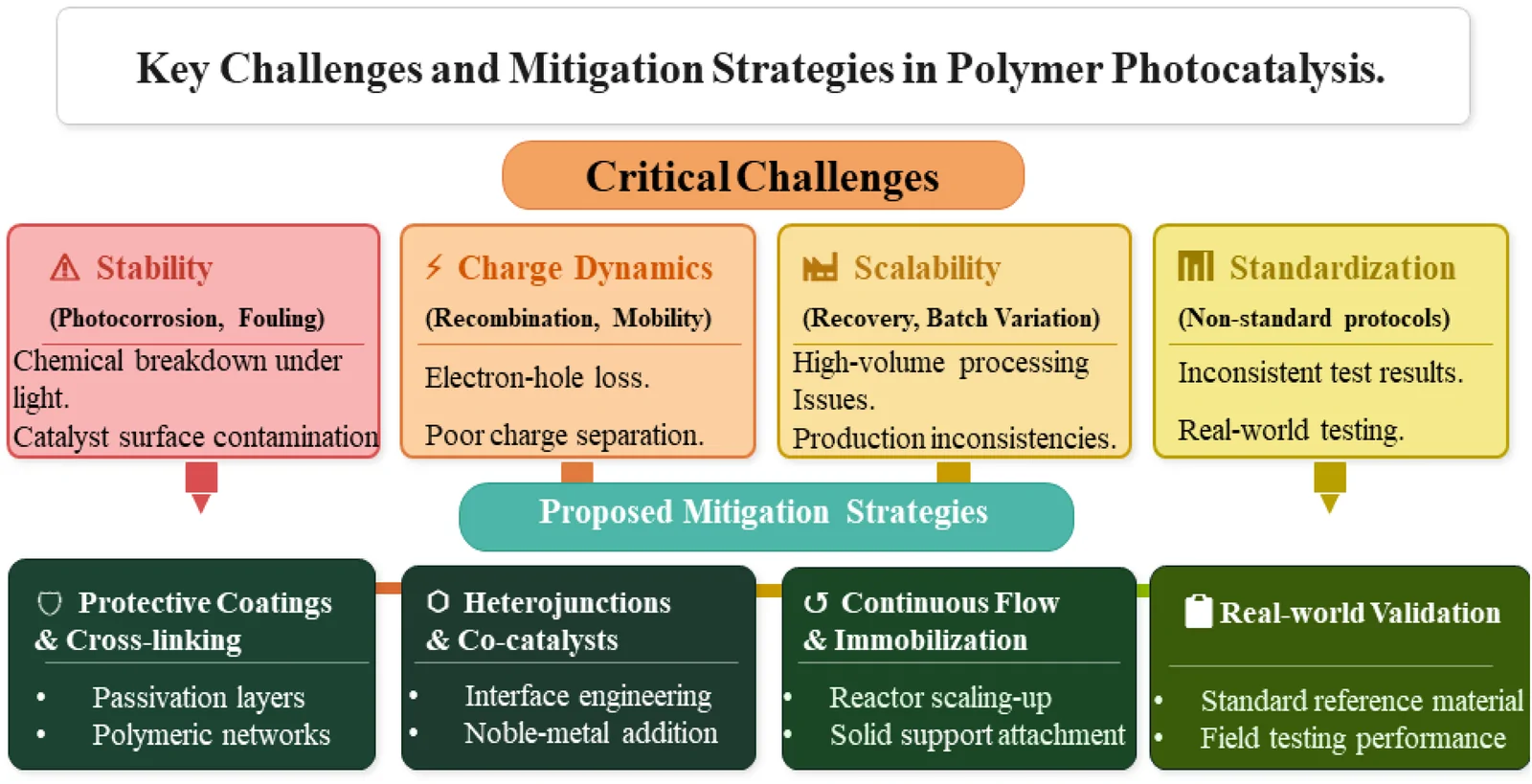
Key challenges and mitigation strategies in polymer photocatalysis.

The self-oxidation or reduction under prolonged light irradiation, adsorption of non-degradable byproducts can passivate the catalyst surface; heavy metal ions or specific organic molecules can act as poisons, the exothermic nature of some reactions can lead to changes in the catalyst's crystallinity, suspension-based products, and physical abrasion are the major problems encountered during the mechanism.^7,169^ In addition to the operational problems, there are fundamental limitations of the polymer photocatalysts, such as a low dielectric constant, which results in exciton binding in the polymer (Fig. 11(a)), an amorphous phase with limited charge transfer (Fig. 11(b)) and trace amounts of metals that can serve as recombination centers (Fig. 11(c)). To overcome the above limitations, three complementary strategies have evolved, involving molecular design to tailor electronic properties, morphology control for boosted activity, and mechanistic understanding through advanced spectroscopies (Fig. 11(b–d)).^169^

**Fig. 11:**
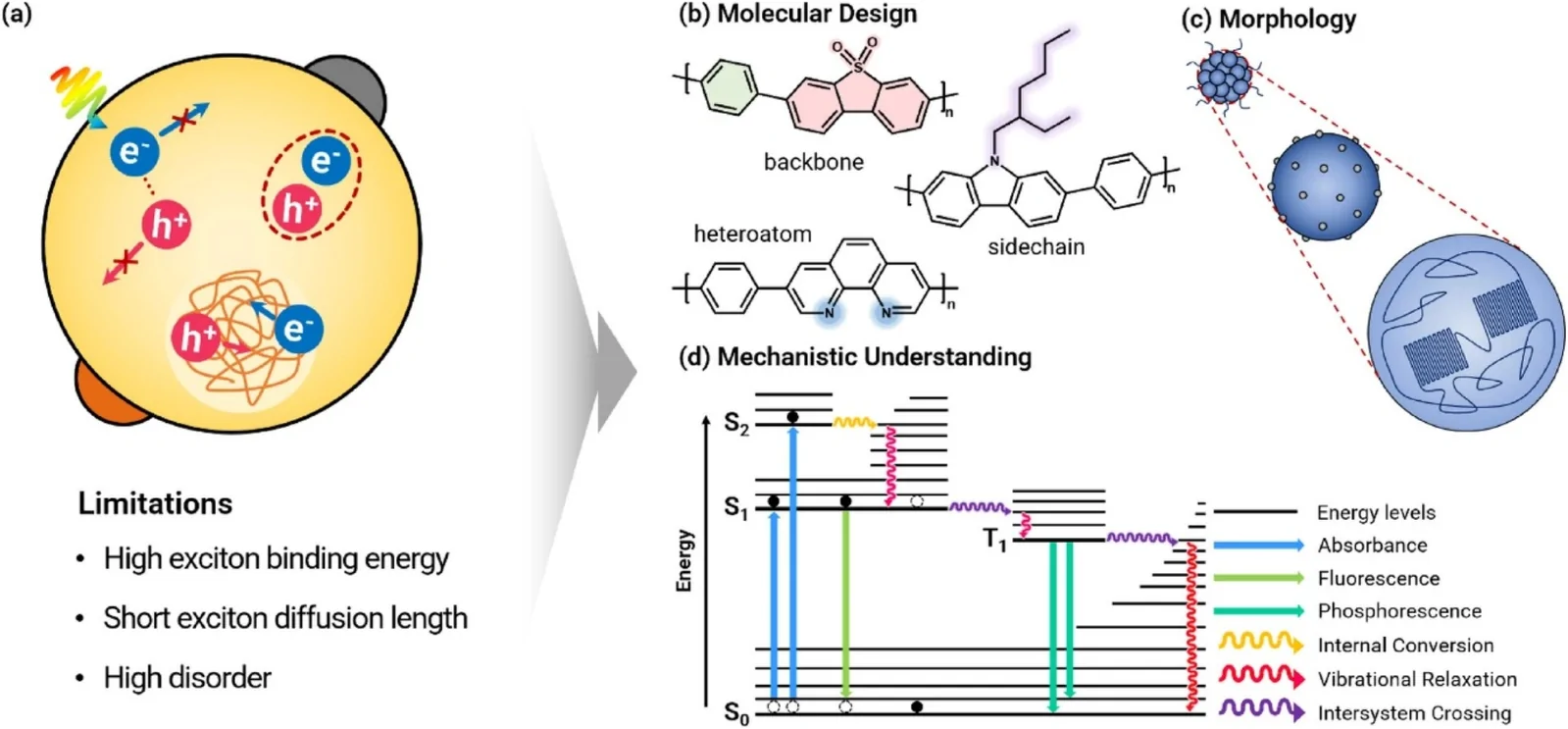
Intrinsic limitation of organic photocatalysts and ways of overcoming them. (a) Low dielectric constants, exciton binding, amorphous morphology, and trace impurities are the key challenges. (b) Molecular designing approaches. (c) Analyze and discuss the methods of morphology control. (d) Advanced spectroscopic methods, mechanistic understanding. Reprinted from, ref. 169*Nano Convergence*, 2026.

The difference between recyclability and real long-term stability in photocatalytic environmental remediation is one of the prominent issues.^170^ The long-term stability and reusability of the heterogeneous photocatalysts are also important but not well-studied issues, as mentioned by recent review articles.^170^ The assessment of stability should then not be made on the basis of short reusability, but also on the basis of run time, operational stability and material stability. A stable photocatalyst should be able to operate normally without any change of crystal structure or chemical formula during the lifetime of the photocatalyst, and it must be accompanied by the post-test structural characterization of the photocatalyst.^171,172^

Stability should be considered as a multi-dimensional property. The deactivation may result from intrinsic instability of the material or from instability caused by photo/redox reactions under operating conditions. One of the important mechanisms isphotocorrosion in which the photons generate electrons or holes that either reduce or oxidize the catalyst itself, instead of the desired reaction.^171^ Long-term testing should detect unstable factors and deactivation mechanisms, not just apparent durability. The revised manuscript therefore separates chemical and photochemical stability from reuse performance. Carbon nitride photocatalysts, for example, have been shown to deactivate through Pt co-catalyst detachment driven by the combination of photocorrosion and mechanical stirring. Both solution composition and the physical environment (light intensity, oxygen, temperature) influence deactivation pathways and should be taken into account along with the catalyst composition.^171,173^ Activity enhancement by surface modification can lead to cytotoxicity, and noble metal co-catalysts can leach under the operating conditions, resulting in secondary environmental hazards.^152^ Although, noble metal-free systems overcome the drawbacks associated with noble metals, practical issues like photocatalyst recovery, mass-transfer limitations, and reactor design still exist.^174^

An important aspect of practical implementation is that green cannot be assumed solely from a metal-free or bio-derived composition.^152,174^ Evaluating the greenness of photocatalytic processes based on the composition of the catalyst is misleading, because excess reagent, toxic solvents, and specially prepared starting materials can be used. The sustainability assessment should include the entire protocol, including the preparation of the catalyst, work-up and purification, and should include quantitative and qualitative considerations. Better, safer and more sustainable synthetic routes are required for scale up, especially for the use of organic solvents and purification procedures.^152^ This is particularly important when it comes to choosing solvents, since semiconductor photocatalysis typically takes place in the condensed phase where the volume of the reaction mixture is dominated by the solvent and the use of the solvent for photocatalysis contributes significantly to the energy consumption, waste generation and emissions. The cost of photocatalytic systems has been found to be affected more by the design of the reactors and the amount of energy supplied to the system than by the composition of the catalyst.^174^ The operational energy of the light source is therefore a critical but often overlooked in sustainability assessments. A recurring limitation in the polymer-photocatalysis literature is the treatment of the light source as a free input. Photocatalytic systems can be a significant contributor to the operating cost and life-cycle environmental impact for artificial illumination when electricity is a significant component. For water treatment using UV-LED photocatalytic technology, the most important environmental hotspots have been identified as electricity consumption and production of immobilized catalyst.^153^ The environmental impacts have been reduced by 55% for a suspended catalyst and 15% for an immobilized system when switching to a renewable electricity mix.^153^ The choice of reactor or light source can also have a significant impact on operating expense. One comparison of four photo-reactor configurations found the cost of the solar compound parabolic collector to be lowest per liter treated and per mg of pollutant removed, while the lowest cost per ppm removed was obtained from an artificial black-light-blue lamp.^175^ The electrical energy per order is a widely used metric for the electrical energy required to reduce contaminant concentration by one order of magnitude per unit volume, formalized for advanced oxidation technologies.^176^ Numerous reported polymer-photocatalysis studies lack adequate information on electrical power or *E*_EO_, making it difficult to quantitatively compare energy performance of different systems. However, a recent systematic review revealed that only 0.09% of photocatalysis studies published explicitly refer to techno-economic or life-cycle assessment, and the few studies available consistently point to high electricity consumption in UV-driven photocatalytic systems and to impacts on the catalyst synthesis as major hurdles to scale-up photocatalysis.^177^ Therefore, future works are suggested to study at least lamp electrical power, delivered irradiance, irradiation time and *E*_EO_ if kinetic information is available.

Recent studies suggest the selection of solvents should incorporate Environment, Health, and Safety (EHS) screening, Life Cycle Assessment (LCA) and, at a practical scale, Techno-Economic Analysis (TEA).^174,178^ Thus, future studies on the evaluation of polymer photocatalysts should go beyond composition-based greenness and follow a full sustainability approach including assessment of the catalyst lifetime, recovery pathways, immobilization methods, material criticality, process energy consumption, solvent selection and life-cycle impacts.^152^ Long duration failure modes and leaching issues are not addressed by the stability data reported in the literature (usually 3–5 recycle runs).^170,171^

The light source degradation and mass transfer limitations can fluctuate the reaction environment, affecting the stability and durability of the photocatalytic process in such a way that can lead to changes in the redox potentials of the photogenerated charges, the rate of photocatalytic activity, and concentration gradients of reactants and products. Moreover, the design of the photocatalytic reactor also plays a crucial role in stability.^179^ Affecting the photocatalytic efficiency, the band gap, insufficient light penetration, and rapid electron–hole recombination are the major factors that limit the overall energy conversion efficiency.^180^ In addition to ineffective charge separation, slow surface reactions may lead to charge accumulation and potentially catalyst deactivation. The limited number of active sites, selection, and stability of the photocatalyst impact the overall efficiency in terms of target product formation. Suboptimal operational conditions, where factors such as temperature, pH, and reactant concentration can significantly influence the photocatalytic efficiency. Identifying and maintaining optimal conditions can be challenging. Various effective mitigation measures have been developed to address the above problems.

The trends in polymer-based photocatalysis focus on the improvement of performance, their extension and the improvement of their sustainability. Absorption of visible light, which occurs much more often than absorption of UV, is engineered, and the present research focuses on the design of polymers that efficiently absorb a greater fraction of the solar spectrum. This can be realized by either the inclusion of certain chromophores, the extension of the conjugated polymer backbone or the creation of donor–acceptor structures with a reduced band gap.^136^ Knowing how to hybridize with nanomaterials to achieve better absorption in this way, polymer has been incorporated with nanomaterials such as plasmonic nanoparticles (*e.g.*, gold and silver) which have the property of surface plasmon resonance for better absorption of the visible light. Likewise, upconversion nanoparticles are being incorporated, which are designed to convert near-infrared light to UV or visible light and be absorbed by the polymer. Additionally, quantum dots and carbon dots are being investigated for their special light emission capabilities which could further improve the use of light.^138^

Advanced conjugated polymer architectures will be engineered to include precisely controlled architectures that allow for the efficient generation, separation and transport of the photogenerated electrons and holes with little recombination. This has included the development of polymers with embedded electric fields or alignments of the energy levels.^139,140^ This is the way heterojunctions are built between polymers made of different semiconductors or between polymers and inorganic semiconductors (such as TiO_2_, ZnO) and the formation of heterojunctions is one of the increasing trends.^141–146^ This separation of charges can occur through these interfaces, as they allow electrons and holes to move to other materials. Another approach is to follow the natural photosynthesis pathway in order to achieve more efficient separation of charges and higher redox potentials (Z-scheme systems). Co-catalysts (*e.g.*, noble metals, metal oxides, molecular catalysts) are also being added to or onto the polymer photocatalyst to promote the transfer of charge to the reactants and decrease the activation energy for the surface reactions.^147^ The convergence of these trends suggests a promising and exciting future for polymer-based photocatalysis, as researchers continue to develop innovative materials and methods with enhanced efficiencies, stabilities, and capabilities for various environmental and energy applications.

### 7.1 Critical synthesis of current understanding

Heterojunctions of Type-II, Z-scheme, or S-scheme are widely claimed to improve charge separation through band alignment. Yet the same material system is often assigned different mechanisms across studies. This discrepancy typically reflects the indirect evidence used, such as PL quenching or radical trapping, rather than a real difference in mechanism.^28,31,32^ The surface area is often assumed to scale with activity. However, some studies show bulk or mesoporous morphologies outperforming high-surface-area nanosheets. This is likely due to diffusion limits in micropores and uncontrolled defect density.^77,78,110–113^ Metal-free or bio-derived composition is often equated with sustainability. Yet many such routes use hazardous solvents or precursors. The ‘green’ is therefore being judged by composition alone, rather than by full life-cycle assessment.^84,86,170^ High degradation percentages are often equated with remediation. However, TOC and LC-MS/MS analyses show incomplete mineralization. Some transformation products are more toxic than the parent compound.^141,143,145,151,153^ The correlation between molecular weight, dispersity, sequence order, and defect density with performance remains uncharacterized. A detailed sustainability assessment over the full life cycle is also still lacking.^181^

## 8 Future outlook and research trajectory

The new research initiatives to take polymer-based photocatalysis from academia to industry require a paradigm shift towards stability engineering and system optimization. One of the major challenges is the creation of intrinsically photostable frameworks – many of the current polymers are subject to chemical degradation under high flux radiation. It is important to continue to design systems with rigid, conjugated backbones, such as covalent triazine frameworks (CTFs) and poly-heptazine imides.^4^ At the same time, real-time spectroscopic investigations, such as transient absorption spectroscopy, are crucial to understand the degradation pathways and to increase the lifetime of a catalyst. An important aspect to tackle performance bottlenecks in hybrid systems is the design of their interfaces.^169^ It is desirable to develop atomically precise heterojunctions instead of just mixing materials physically, and methods like van der Waals assembly and epitaxial deposition are in development which will help to reduce the interfacial resistance. This structural improvement needs to be in parallel with the standardization of the photocatalytic performance metrics.^180^ At present, quantum yields and reaction rates are reported differently across studies, making it difficult to compare between studies. For new materials, universal protocols, including AQY under monochromatic light, are critically needed to compare them to the state of the art.^154,161^

The chemical space of conjugated polymers is enormous and can be explored more efficiently by using computational innovation. Machine learning-driven discovery is now essential, where the power of machine learning can give predictions of how different substituent groups will affect the redox potentials, before investing in time-consuming synthesis.^171,180^ In addition, the field should break the cycle of too many electron donors being sacrificed. To realize an actual artificial photosynthesis, it is necessary to go beyond the use of different reagents such as TEOA or methanol and to split water and directly reduce CO_2_ with water as the electron donor. This is difficult, but is still a basis for sustainable fuel production.^154,161^ Research should focus on high-value applications where polymer photocatalysts hold a competitive advantage. These include selective degradation of persistent micropollutants, N_2_ activation for nitrogen fixation, and complex plastic upcycling.^181^ Polymer photocatalysis is at a critical juncture. Moving beyond ‘performance-first’ reporting toward mechanism-driven, rigorous analysis will determine whether these materials remain confined to the laboratory or emerge as pillars of the circular economy. The success in industrial adoption will ultimately hinge on three factors: mechanistic rigour, interfacial precision, and techno-economic realism.^169^

## 9 Summary

Polymer-based photocatalysis has become a transformative platform for environmental remediation and renewable energy conversion in a sustainable manner, and has been known for having several advantages over conventional inorganic photocatalysts. In this review, the fundamental concepts, synthesis methods, material structures, performance conditions, and fields of application of advanced photocatalytic systems have been discussed. Various synthesis options allow tailoring of morphology, porosity, and surface functionality. The polymer photocatalysts can be divided into the conducting polymers, the conjugated polymers, the metal-free frameworks and the emerging architectures, all of which have their own advantages for specific applications. A critical evaluation reveals that Type-II, Z-scheme, and S-scheme mechanisms are widely claimed for charge separation in heterojunctions, but these mechanisms require more rigorous investigation. The limitation of low dielectric constant prevents the generation of free charge, insufficient long-term photostability, dependence on electron donors which are sacrificed, which makes the process economically unfeasible, and the absence of a standardized testing protocol are key challenges. The future research aims include rational design of heterojunction interface, elimination of sacrificial agents, design of inherently photostable architectures, computational acceleration by machine learning, and integrated techno-economic assessments. The photocatalytic properties of polymers are at an inflection point – the future of the technology hinges on mechanisms, interfaces and eco-friendly design for the circular economy.

## Conflicts of interest

The authors declare that they have no known competing financial interests or personal relationships that could have appeared to influence the work reported in this paper.

## Data Availability

Data sharing is not applicable to this article as no new datasets were generated or analyzed during the current study. All relevant data and literature sources utilized in this review are available within the text and the comprehensive reference list.

## References

[cit1] Ong W. J. (2016). *et al.*, Graphitic Carbon Nitride (g-C_3_N_4_)-Based Photocatalysts for Artificial Photosynthesis and Environmental Remediation: Are We a Step Closer to Achieving Sustainability?. Chem. Rev..

[cit2] Xu S. (2019). *et al.*, A nitrogen-rich 2D sp^2^-carbon-linked conjugated polymer framework as a high-performance cathode for lithium-ion batteries. Angew. Chem..

[cit3] Augustin A. (2022). *et al.*, Recent development of organic–inorganic hybrid photocatalysts for biomass conversion into hydrogen production. Nanoscale Adv..

[cit4] Wang H. (2020). *et al.*, Covalent Organic Framework Photocatalysts: Structures and Applications. Chem. Soc. Rev..

[cit5] Banerjee T. (2021). *et al.*, Polymer photocatalysts for solar-to-chemical energy conversion. Nat. Rev. Mater..

[cit6] Dai C., Liu B. (2020). Conjugated polymers for visible-light-driven photocatalysis. Energy Environ. Sci..

[cit7] Wang Y. (2019). *et al.*, Current understanding and challenges of solar-driven hydrogen generation using polymeric photocatalysts. Nat. Energy.

[cit8] Balan A., Baran D., Toppare L. (2011). Benzotriazole containing conjugated polymers for multipurpose organic electronic applications. Polym. Chem..

[cit9] Bin H. (2016). *et al.*, 11.4% Efficiency non-fullerene polymer solar cells with trialkylsilyl substituted 2D-conjugated polymer as donor. Nat. Commun..

[cit10] Mohan B. (2025). *et al.*, Patterning and dynamics of structured surfaces in Metal/Covalent organic frameworks and polymer chemistry. Top. Curr. Chem..

[cit11] Zhang G. (2017). *et al.*, Optimizing optical absorption, exciton dissociation, and charge transfer of a polymeric carbon nitride with ultrahigh solar hydrogen production activity. Angew. Chem..

[cit12] Li N., Ma Y., Sun W. (2024). Exploring the Dynamics of Charge Transfer in Photocatalysis: Applications of Femtosecond Transient Absorption Spectroscopy. Molecules.

[cit13] Melinte V., Stroea L., Chibac-Scutaru A. L. (2019). Polymer Nanocomposites for Photocatalytic Applications. Catalysts.

[cit14] Bariki R. (2024). *et al.*, The Development of Metal-Free Porous Organic Polymers for Sustainable Carbon Dioxide Photoreduction. Nanomaterials.

[cit15] Ru C. (2025). *et al.*, Regulation of Exciton Effects in Functionalized Conjugated Polymers by B–N Lewis Pairs for Visible-Light Photocatalysis. Angew. Chem..

[cit16] Wang H. (2020). *et al.*, Excitonic effects in polymeric photocatalysts. Angew. Chem..

[cit17] Ru C. (2026). *et al.*, Regulating Exciton Dynamics in Organic Polymer Photocatalysts with Dual-Polarization Structure. Adv. Funct. Mater..

[cit18] Wang H. (2017). *et al.*, Insights into the excitonic processes in polymeric photocatalysts. Chem. Sci..

[cit19] Choi J. (2024). *et al.*, Understanding charge carrier dynamics in organic photocatalysts for hydrogen evolution. Energy Environ. Sci..

[cit20] Ouyang L. (2026). *et al.*, Tuning Exciton–Phonon Coupling by Isotope Engineering: Regulated Charge Carrier Evolution in Carbon Nitride Photocatalysts. Angew. Chem..

[cit21] Xiao J. (2022). *et al.*, Rational design of alkynyl-based linear donor–π–acceptor conjugated polymers with accelerated exciton dissociation for photocatalysis. Macromolecules.

[cit22] Sachs M. (2020). *et al.*, Tracking charge transfer to residual metal clusters in conjugated polymers for photocatalytic hydrogen evolution. J. Am. Chem. Soc..

[cit23] Wang W. (2025). *et al.*, Exciton Engineering in Organic Photocatalysis: Mechanisms and Applications. Environ. Funct. Mater..

[cit24] Chen H. (2022). *et al.*, Engineering covalent organic frameworks as heterogeneous photocatalysts for organic transformations. Angew. Chem., Int. Ed..

[cit25] Kaur B. (2025). *et al.*, Catalytic performance of graphdiyne for CO_2_ reduction and charge dynamics progress. J. Environ. Chem. Eng..

[cit26] Wang L. (2024). *et al.*, Molecular-level regulation strategies toward efficient charge separation in donor–acceptor type conjugated polymers for boosted energy-related photocatalysis. Adv. Energy Mater..

[cit27] Hasnan N. S. N., Nordin N. A., Mohamed M. A. (2024). Synergistic interaction and hybrid association of conducting polymer photocatalysts/photoelectrodes for emerging visible light active photocatalytic applications. J. Mater. Chem. A.

[cit28] Sayed M. (2026). *et al.*, S-scheme shapes heterojunction photocatalysis. Acc. Chem. Res..

[cit29] Cheng Q. (2026). *et al.*, Core–shell CoTiO_3_@SnS_2_ S-scheme heterojunction photocatalysts enabling highly selective CO_2_ photoreduction. J. Mater. Sci. Technol..

[cit30] Sun L. (2012). *et al.*, Enhanced visible-light photocatalytic activity of g-C_3_N_4_-ZnWO_4_ by fabricating a heterojunction: investigation based on experimental and theoretical studies. J. Mater. Chem..

[cit31] Sudrajat H., Nobatova M. (2025). Heterojunction photocatalysts: where are they headed?. RSC Appl. Interfaces.

[cit32] Jung W. (2025). *et al.*, Photocatalytic Hydrogen Evolution with Conjugated Polymers: Structure–Property Insights and Design Strategies. Adv. Energy Mater..

[cit33] Zhang J. (2024). *et al.*, Recent progress on S-scheme heterojunction strategy enabling polymer carbon nitrides C_3_N_4_ and C_3_N_5_ enhanced photocatalysis in energy conversion and environmental remediation. Curr. Opin. Chem. Eng..

[cit34] Sardar S. (2015). *et al.*, Enhanced charge separation and FRET at heterojunctions between semiconductor nanoparticles and conducting polymer nanofibers for efficient solar light harvesting. Sci. Rep..

[cit35] Zha R. (2026). *et al.*, Design principles and interface engineering of the organic–inorganic hybrid S-scheme heterojunctions for advancing photocatalysis. Adv. Compos. Hybrid Mater..

[cit36] Palliyalil S., Poovathumkuzhi N. C., Vigneshwaran S. (2026). Mechanistic insights into Type-I and Type-II MoS∼2∼ heterojunctions for the photodegradation of organic dyes. Discover Green Chem..

[cit37] Moniz S. J. (2015). *et al.*, Visible-light driven heterojunction photocatalysts for water splitting-a critical review. Energy Environ. Sci..

[cit38] Markhabayeva A. A. (2023). *et al.*, Construction of a ZnO heterogeneous structure using Co_3_O_4_ as a co-catalyst to enhance photoelectrochemical performance. Materials.

[cit39] Yu L. (2016). *et al.*, Highly efficient Bi_2_O_2_CO_3_/BiOCl photocatalyst based on heterojunction with enhanced dye-sensitization under visible light. Appl. Catal., B.

[cit40] Liu B. (2019). *et al.*, Construction of a novel Z-scheme heterojunction with molecular grafted carbon nitride nanosheets and V_2_O_5_ for highly efficient photocatalysis. J. Phys. Chem. C.

[cit41] Hmud K. (2025). *et al.*, Advanced automated dip-coating strategy for high-performance WO_3_/BiVO_4_ photoanodes in durable photoelectrocatalytic water splitting and organic dye degradation. Int. J. Hydrogen Energy.

[cit42] Liao G. (2010). *et al.*, Photonic crystal coupled TiO_2_/polymer hybrid for efficient photocatalysis under visible light irradiation. Environ. Sci. Technol..

[cit43] Zhang F. (2023). *et al.*, Electrospinning photocatalysis meet in situ irradiated XPS: recent mechanisms advances and challenges. Small.

[cit44] Neshastehgar M., Jamshidi M., Ghamarpoor R. (2025). Self-assembly TiO_2_@ Silane@ SiO_2_ core–shell as s-scheme heterojunction photocatalyst against methylene blue degradation: synthesis and mechanism insights. J. Mol. Struct..

[cit45] Lopez-Calixto C. G. (2020). *et al.*, Hybrids based on BOPHY-conjugated porous polymers as photocatalysts for hydrogen production: insight into the charge transfer pathway. ACS Catal..

[cit46] Reddy N. L. , *et al.*, Graphitic carbon nitride-based nanocomposite materials for photocatalytic hydrogen generation, in Nanostructured, Functional, and Flexible Materials for Energy Conversion and Storage Systems, Elsevier, 2020, pp. 293–324

[cit47] Chen Y., Jiang D. (2024). Photocatalysis with Covalent Organic Frameworks. Acc. Chem. Res..

[cit48] Liu J. (2023). *et al.*, One-Step Electropolymerization of a Dicyanobenzene-Carbazole-Imidazole Dye to Prepare Photoactive Redox Polymer Films. Polymers.

[cit49] Yang X., Loos J. (2007). Toward high-performance polymer solar cells: The importance of morphology control. Macromolecules.

[cit50] Goddard J. M., Hotchkiss J. H. (2007). Polymer surface modification for the attachment of bioactive compounds. Prog. Polym. Sci..

[cit51] Rosman N. N. (2022). *et al.*, An overview of co-catalysts on metal oxides for photocatalytic water splitting. Int. J. Hydrogen Energy.

[cit52] Enesca A., Cazan C. (2022). Polymer composite-based materials with photocatalytic applications in wastewater organic pollutant removal: a mini review. Polymers.

[cit53] Shen Y. (2024). *et al.*, Photocatalytic Surface Initiation for Area-Selective Chemical Vapor Deposition of Polymer Thin Film. ACS Mater. Lett..

[cit54] Pellegrino G. (2018). *et al.*, Polymeric platform for the growth of chemically anchored ZnO nanostructures by ALD. RSC Adv..

[cit55] Pavasupree S., Dubas S. T., Rangkupan R. (2015). Surface modification of polypropylene non-woven fibers with TiO_2_ nanoparticles via layer-by-layer self assembly method: Preparation and photocatalytic activity. J. Environ. Sci..

[cit56] Mishra B. (2019). *et al.*, Engineering the Surface of a Polymeric Photocatalyst for Stable Solar-to-Chemical Fuel Conversion from Seawater. ChemSusChem.

[cit57] Riaz U., Ashraf S., Kashyap J. (2015). Role of conducting polymers in enhancing TiO_2_-based photocatalytic dye degradation: a short review. Polym.-Plast. Technol. Eng..

[cit58] Matias M. L. (2023). *et al.*, Microwave synthesis of visible-light-activated g-C_3_N_4_/TiO_2_ photocatalysts. Nanomaterials.

[cit59] Li Y. (2015). *et al.*, Seed-induced growing various TiO_2_ nanostructures on g-C_3_N_4_ nanosheets with much enhanced photocatalytic activity under visible light. J. Hazard. Mater..

[cit60] Kuldeep A., Dhabbe R., Garadkar K. (2021). Development of g-C_3_N_4_-TiO_2_ visible active hybrid photocatalyst for the photodegradation of methyl orange. Res. Chem. Intermed..

[cit61] Briševac D. (2025). *et al.*, Influence of Different Precursors on Properties and Photocatalytic Activity of g-C_3_N_4_ Synthesized via Thermal Polymerization. Materials.

[cit62] Prajapati P. K., Saini S., Jain S. L. (2020). Nickel mediated palladium free photocatalytic Suzuki-coupling reaction under visible light irradiation. J. Mater. Chem. A.

[cit63] Cui Y. (2011). *et al.*, Synthesis of bulk and nanoporous carbon nitride polymers from ammonium thiocyanate for photocatalytic hydrogen evolution. J. Mater. Chem..

[cit64] Yu M. (2021). *et al.*, Engineering nanoparticulate organic photocatalysts via a scalable flash nanoprecipitation process for efficient hydrogen production. Angew. Chem., Int. Ed..

[cit65] Li Y. (2011). *et al.*, Preparation of Ag-doped TiO_2_ nanoparticles by a miniemulsion method and their photoactivity in visible light illuminations. Mater. Chem. Phys..

[cit66] Zhang Z., Zuo F., Feng P. (2010). Hard template synthesis of crystalline mesoporous anatase TiO_2_ for photocatalytic hydrogen evolution. J. Mater. Chem..

[cit67] Yang W. (2012). *et al.*, Microwave-assisted synthesis of porous Ag_2_S–Ag hybrid nanotubes with high visible-light photocatalytic activity. Angew. Chem., Int. Ed..

[cit68] Zhong B. (2023). *et al.*, Advances in ultrasound-assisted photocatalyst synthesis and piezo-photocatalysts. J. Mater. Chem. A.

[cit69] Lonkar S. P., Pillai V., Abdala A. (2019). Solvent-free synthesis of ZnO-graphene nanocomposite with superior photocatalytic activity. Appl. Surf. Sci..

[cit70] Zhang P. (2023). *et al.*, Elemental-Doping-Induced Orbital Redistribution in Conjugated Polymer Boosts Charge-Transfer-Mediated Singlet Oxygen Production. J. Phys. Chem. Lett..

[cit71] Zuo C. (2023). *et al.*, S-scheme 2D/2D heterojunction of ZnTiO_3_ nanosheets/Bi2WO_6_ nanosheets with enhanced photoelectrocatalytic activity for phenol wastewater under visible light. Molecules.

[cit72] Zhang H. (2015). *et al.*, Organic–Inorganic hybrid materials based on basket-like {Ca⊂P_6_Mo_18_O_73_} cages. Inorg. Chem..

[cit73] Yifan H. (2025). *et al.*, P3HT/g-C_3_N_4_ composite fiber membranes for high-performance photocatalytic hydrogen evolution. Appl. Surf. Sci..

[cit74] Meng F. (2025). *et al.*, Diffusion-controlled charge separation in conjugated polymer heterojunctions for visible light-driven hydrogen production. Sep. Purif. Technol..

[cit75] Chengxiong X. (2026). *et al.*, Fabrication of S-scheme S-C_3_N_4_/Bi_2_WO_6_ heterojunctions with interfacial oxygen and nitrogen dual vacancies for enhanced photocatalytic activity. J. Environ. Chem. Eng..

[cit76] Lee S. L., Chang C.-J. (2019). Recent developments about conductive polymer based composite photocatalysts. Polymers.

[cit77] Pavliuk M. V. (2022). *et al.*, Preparation, characterization, evaluation and mechanistic study of organic polymer nano-photocatalysts for solar fuel production. Chem. Soc. Rev..

[cit78] Bohra H. (2018). *et al.*, “Greener” and modular synthesis of triazine-based conjugated porous polymers via direct arylation polymerization: structure–function relationship and photocatalytic application. Polym. Chem..

[cit79] Zhu Q. (2025). *et al.*, Resorcinarene as a resin-like monomer for the synthesis of polymer photocatalysts with anisotropic morphology toward improved H_2_O_2_ photosynthesis. Chem. Eng. J..

[cit80] D'Arienzo M. (2011). *et al.*, Photogenerated defects in shape-controlled TiO_2_ anatase nanocrystals: a probe to evaluate the role of crystal facets in photocatalytic processes. J. Am. Chem. Soc..

[cit81] Kröger J. (2021). *et al.*, Morphology control in 2D carbon nitrides: impact of particle size on optoelectronic properties and photocatalysis. Adv. Funct. Mater..

[cit82] Baig U. (2017). *et al.*, Band gap engineered polymeric–inorganic nanocomposite catalysts: Synthesis, isothermal stability, photocatalytic activity and photovoltaic performance. J. Mater. Sci. Technol..

[cit83] Liu X., Cai L. (2018). Novel indirect Z-scheme photocatalyst of Ag nanoparticles and polymer polypyrrole co-modified BiOBr for photocatalytic decomposition of organic pollutants. Appl. Surf. Sci..

[cit84] Zhang Z., Wang W., Gao E. (2014). Polypyrrole/Bi_2_WO_6_ composite with high charge separation efficiency and enhanced photocatalytic activity. J. Mater. Sci..

[cit85] Yan B. (2017). *et al.*, Flexible photocatalytic composite film of ZnO-microrods/polypyrrole. ACS Appl. Mater. Interfaces.

[cit86] El-Bery H. M. (2021). *et al.*, Efficient non-metal based conducting polymers for photocatalytic hydrogen production: comparative study between polyaniline, polypyrrole and PEDOT. RSC Adv..

[cit87] El-Bery H. M. (2021). *et al.*, Efficient non-metal based conducting polymers for photocatalytic hydrogen production: comparative study between polyaniline, polypyrrole and PEDOT. RSC Adv..

[cit88] Fu Y., Janczarek M. (2025). UV and visible light-induced photocatalytic efficiency of polyaniline/titanium dioxide heterostructures. Molecules.

[cit89] Gong H. (2024). *et al.*, Functionalized Linear Conjugated Polymer/TiO_2_ Heterojunctions for Significantly Enhancing Photocatalytic H2 Evolution. Molecules.

[cit90] Zheng Y. (2022). *et al.*, Triptycene incorporated carbon nitride based donor–acceptor conjugated polymers with superior visible-light photocatalytic activities. J. Colloid Interface Sci..

[cit91] Song H. (2022). *et al.*, Synergy of intermolecular Donor–Acceptor and ultrathin structures in crystalline carbon nitride for efficient photocatalytic hydrogen evolution. J. Colloid Interface Sci..

[cit92] Yu F. (2021). *et al.*, Construction of polymeric carbon nitride and dibenzothiophene dioxide-based intramolecular donor–acceptor conjugated copolymers for photocatalytic H_2_ evolution. Nanoscale Adv..

[cit93] Liu F. (2022). *et al.*, Regulating excitonic effects in covalent organic frameworks to promote free charge carrier generation. ACS Catal..

[cit94] Wang Y. (2022). *et al.*, Facile construction of fully sp^2^-carbon conjugated two-dimensional covalent organic frameworks containing benzobisthiazole units. Nat. Commun..

[cit95] Li C. (2022). *et al.*, Covalent organic frameworks with high quantum efficiency in sacrificial photocatalytic hydrogen evolution. Nat. Commun..

[cit96] Liu B. (2025). *et al.*, *In Situ* Crystalline Growth ZnS Nanoparticles on Conjugated Polymer for Enhancement of the Photocatalytic Performance. Polymers.

[cit97] Wang X. (2009). *et al.*, A metal-free polymeric photocatalyst for hydrogen production from water under visible light. Nat. Mater..

[cit98] Wu W. (2025). *et al.*, Metal-free polymer photocatalysts for efficient gas-phase reduction of atmospheric CO2 and simultaneous H_2_O_2_ production. Green Chem..

[cit99] Kou M. (2021). *et al.*, Photocatalytic CO_2_ conversion over single-atom MoN_2_ sites of covalent organic framework. Appl. Catal., B.

[cit100] Das R. (2023). *et al.*, Engineering the charge density on an In2.77S4/porous organic polymer hybrid photocatalyst for CO_2_-to-ethylene conversion reaction. J. Am. Chem. Soc..

[cit101] Skorjanc T. (2022). *et al.*, Polythiacalixarene-embedded gold nanoparticles for visible-light-driven photocatalytic CO2 reduction. ACS Appl. Mater. Interfaces.

[cit102] Zhang H. (2016). *et al.*, Efficient visible-light-driven carbon dioxide reduction by a single-atom implanted metal–organic framework. Angew. Chem..

[cit103] Otgonbayar Z. (2021). *et al.*, Novel ternary composite of LaYAgO_4_ and TiO_2_ united with graphene and its complement: Photocatalytic performance of CO_2_ reduction into methanol. Mater. Sci. Semicond. Process..

[cit104] Hayat A. (2023). *et al.*, Recent advances in ground-breaking conjugated microporous polymers-based materials, their synthesis, modification and potential applications. Mater. Today.

[cit105] Righetti G. I. C., Faedi F., Famulari A. (2024). Embracing Sustainability: The World of Bio-Based Polymers in a Mini Review. Polymers.

[cit106] Zhu A. (2020). *et al.*, Dopant-induced edge and basal plane catalytic sites on ultrathin C3N4 nanosheets for photocatalytic water reduction. ACS Sustainable Chem. Eng..

[cit107] Wei S., Zhang G., Xu X. (2018). Activating BaTaO_2_N by Ca modifications and cobalt oxide for visible light photocatalytic water oxidation reactions. Appl. Catal., B.

[cit108] Wang C. (2021). *et al.*, Ultrathin Crystalline Covalent-Triazine-Framework Nanosheets with Electron Donor Groups for Synergistically Enhanced Photocatalytic Water Splitting. Angew. Chem., Int. Ed..

[cit109] Ibad M. F. (2017). *et al.*, Impact of the crystallinity of mesoporous polymeric graphitic carbon nitride on the photocatalytic performance under UV and visible light. Microporous Mesoporous Mater..

[cit110] Willner B. J. (2024). *et al.*, Correlation between the Molecular Properties of Semiconducting Polymers of Intrinsic Microporosity and Their Photocatalytic Hydrogen Production. J. Am. Chem. Soc..

[cit111] Zhang K. (2013). *et al.*, Surface area control and photocatalytic activity of conjugated microporous poly (benzothiadiazole) networks. Angew. Chem..

[cit112] Li X., Masters A. F., Maschmeyer T. (2015). Photocatalytic hydrogen evolution from silica-templated polymeric graphitic carbon nitride-is the surface area important?. ChemCatChem.

[cit113] Zhang G., Lan Z. A., Wang X. (2016). Conjugated polymers: catalysts for photocatalytic hydrogen evolution. Angew. Chem., Int. Ed..

[cit114] Kim S. (2024). *et al.*, Size-Dependent Photocatalytic Reactivity of Conjugated Microporous Polymer Nanoparticles. Adv. Mater..

[cit115] Kumar A., Pandey G. (2017). A review on the factors affecting the photocatalytic degradation of hazardous materials. Mater. Sci. Eng. Int. J..

[cit116] Li S. (2019). *et al.*, Strategies for improving the performance and application of MOFs photocatalysts. ChemCatChem.

[cit117] Vallejo-Rendón D. , *et al.*, Factors affecting photocatalytic activity, in Graphene-Based Photocatalysts for Hydrogen Production and Environmental Remediation, Springer, 2024, pp. 161–180

[cit118] Gusain R., Kumar N., Ray S. S. (2020). Factors influencing the photocatalytic activity of photocatalysts in wastewater treatment. Photocatal. Adv. Oxid. Processes Wastewater Treat..

[cit119] Yang H. (2022). *et al.*, Factors Affecting the Catalytic Performance of Nano-catalysts. Chin. J. Chem..

[cit120] Laxmi R. (2024). *et al.*, Fluorene and Triazine-Based Conjugated Polymer Networks with Tuned Frontier Orbital Energy Levels for Improving Organic Photocatalysis. ACS Appl. Polym. Mater..

[cit121] Ning C.-Z., Dou L., Yang P. (2017). Bandgap engineering in semiconductor alloy nanomaterials with widely tunable compositions. Nat. Rev. Mater..

[cit122] García-Muñoz P. (2022). *et al.*, Photocatalytic degradation of polystyrene nanoplastics in water. A methodological study. J. Environ. Chem. Eng..

[cit123] Sachs M. (2018). *et al.*, Understanding structure–activity relationships in linear polymer photocatalysts for hydrogen evolution. Nat. Commun..

[cit124] Romay M. (2020). *et al.*, Critical issues and guidelines to improve the performance of photocatalytic polymeric membranes. Catalysts.

[cit125] Sánchez-Castro I. (2023). *et al.*, Past, present and future trends in the remediation of heavy-metal contaminated soil-Remediation techniques applied in real soil-contamination events. Heliyon.

[cit126] Bora L. V. , *et al.*, Plastic degradation by photocatalysis: basic concepts and general mechanisms, in Plastic Degradation and Conversion by Photocatalysis (Volume 1): A Sustainable Approach, ACS Publications, 2024, pp. 1–22

[cit127] Queirós J. M. (2025). *et al.*, Photocatalytic and antimicrobial polymer-based hybrid membranes with surface-modified TiO_2_ nanoparticles with 5-aminosalicylic acid and silver nanoparticles. RSC Sustainability.

[cit128] Ubaldi F. (2024). *et al.*, Antimicrobial activity of photocatalytic coatings on surfaces: A systematic review and meta-analysis. Coatings.

[cit129] Hao X. (2017). *et al.*, Preparation of polyaniline modified BiOBr with enhanced photocatalytic activities. Funct. Mater. Lett..

[cit130] Xu Y. (2019). *et al.*, Conjugated conducting polymers PANI decorated Bi_12_O_17_C_l2_ photocatalyst with extended light response range and enhanced photoactivity. Appl. Surf. Sci..

[cit131] Wang Q. (2013). *et al.*, Photodegradation of methyl orange with PANI-modified BiOCl photocatalyst under visible light irradiation. Appl. Surf. Sci..

[cit132] Zhu Y., Li X., Zhu M. (2016). Mesoporous graphitic carbon nitride as photo-catalyst for oxidative desulfurization with oxygen. Catal. Commun..

[cit133] Wang Z. (2018). *et al.*, In situ g-C_3_N_4_ self-sacrificial synthesis of a g-C_3_N_4_/LaCO_3_OH heterostructure with strong interfacial charge transfer and separation for photocatalytic NO removal. J. Mater. Chem. A.

[cit134] Zhang X. (2019). *et al.*, Photocatalytic oxidative desulfurization and denitrogenation of fuels over sodium doped graphitic carbon nitride nanosheets under visible light irradiation. Mater. Chem. Phys..

[cit135] Fontelles-Carceller O. (2016). *et al.*, Interface effects in sunlight-driven Ag/g-C_3_N_4_ composite catalysts: study of the toluene photodegradation quantum efficiency. ACS Appl. Mater. Interfaces.

[cit136] Kong L. (2021). *et al.*, Porous graphitic carbon nitride nanosheets for photocatalytic degradation of formaldehyde gas. Chem. Phys. Lett..

[cit137] Zou Y. (2019). *et al.*, SnO_2_ quantum dots anchored on g-C_3_N_4_ for enhanced visible-light photocatalytic removal of NO and toxic NO_2_ inhibition. Appl. Surf. Sci..

[cit138] Rahimi S. (2014). *et al.*, Photocatalytic removal of cadmium (II) and lead (II) from simulated wastewater at continuous and batch system. Int. J. Environ. Health Eng..

[cit139] Bao L.-L. (2023). *et al.*, A new 2D Zn (II)-based coordination polymer as photocatalyst for photodegradation of methyl orange in water: Effect of photocatalyst dosage and dye concentration. J. Mol. Struct..

[cit140] Zuo W. (2020). *et al.*, Synthesis, structure and photocatalytic dye degradation ability of Co (II)-based coordination polymers. J. Inorg. Organomet. Polym. Mater..

[cit141] Pavel M. (2023). *et al.*, Photocatalytic degradation of organic and inorganic pollutants to harmless end products: assessment of practical application potential for water and air cleaning. Catalysts.

[cit142] Moreira A. J. (2025). *et al.*, Photocatalytic degradation of environmental contaminants: Transformation products and effects on photocatalytic performance. Catalysts.

[cit143] Studziński W. (2025). *et al.*, Environmental and toxicological aspects of sulfamethoxazole photodegradation in the presence of oxidizing agents. Environ. Sci. Pollut. Res..

[cit144] Farzadkia M. (2025). *et al.*, Investigating the toxicity of photocatalytic degradation products from metronidazole using ZnO/polyaniline and TiO_2_/polyaniline nanocomposites: A bioassay study. Desalin. Water Treat..

[cit145] Diamanti M. V. (2025). *et al.*, Toward sustainable photocatalysis: addressing deactivation and environmental impact of anodized and sol–gel photocatalysts. Adv. Sustainable Syst..

[cit146] Zhang Y. (2025). *et al.*, Failure mechanism analysis and emerging strategies for enhancing the photoelectrochemical stability of photoanodes. ChemSusChem.

[cit147] Jamil Q. (2024). *et al.*, Continuous flow photocatalytic reactor for degradation of selected pollutants: Modeling, kinetics, mineralization rate, and toxicity assessment. Heliyon.

[cit148] Evgenidou E. (2026). *et al.*, Beyond degradation: LC-HRMS based tracking of transformation products and toxicity during photocatalytic removal of ceftazidime via advanced oxidation processes. Sustainable Chem. Environ..

[cit149] Jallouli N. (2018). *et al.*, Heterogeneous photocatalytic degradation of ibuprofen in ultrapure water, municipal and pharmaceutical industry wastewaters using a TiO_2_/UV-LED system. Chem. Eng. J..

[cit150] Shi J. (2024). *et al.*, Insights on the efficiency and contribution of single active species in photocatalytic degradation of tetracycline: Priority attack active sites, intermediate products and their toxicity evaluation. J. Environ. Manage..

[cit151] Sdoukou E., Makrelli I., Konstantinou I. (2026). Defect-activated and thermally exfoliated under N2 flow g-C_3_N_4_ photocatalysts for the degradation of ciprofloxacin as a model emerging aquatic pollutant. J. Environ. Chem. Eng..

[cit152] Quintavalla A., Carboni D., Lombardo M. (2024). Green metrics and sustainability in photocatalysis. ChemCatChem.

[cit153] Gowland D. C., Robertson N., Chatzisymeon E. (2024). Life cycle assessment of immobilised and slurry photocatalytic systems for removal of natural organic matter in water. Environments.

[cit154] Rahman M. Z. (2026). *et al.*, Is the use of sacrificial agents a sustainable practice in scalable photocatalytic hydrogen production from water?. Mater. Horiz..

[cit155] Kailasam K. (2013). *et al.*, Room temperature synthesis of heptazine-based microporous polymer networks as photocatalysts for hydrogen evolution. Macromol. Rapid Commun..

[cit156] Kailasam K. (2016). *et al.*, Donor–acceptor-type heptazine-based polymer networks for photocatalytic hydrogen evolution. Energy Technol..

[cit157] Yang L.-F. (2025). *et al.*, Electron–donor engineering of heptazine-based donor–acceptor conjugated microporous polymers for efficient metal-free photocatalytic hydrogen evolution. J. Mater. Chem. A.

[cit158] Liu S. (2024). *et al.*, Tailoring Metal-Ion-Doped Carbon Nitrides for Photocatalytic Oxygen Evolution Reaction. ACS Catal..

[cit159] Du H. (2022). *et al.*, Cu/PCN metal-semiconductor heterojunction by thermal reduction for photoreaction of CO_2_-aerated H_2_O to CH_3_OH and C_2_H_5_OH. ACS Omega.

[cit160] Li X. (2022). *et al.*, High-performance potassium poly(heptazine imide) films for photoelectrochemical water splitting. Chem. Sci..

[cit161] Shi R. (2019). *et al.*, Defect Engineering in Photocatalytic Nitrogen Fixation. ACS Catal..

[cit162] Varala R. (2025). *et al.*, Research Progress of DBU in C–C, C–Heteroatom, and Heteroatom–Heteroatom Bond Formations. Chem. Biodiversity.

[cit163] Rangarajan G., Yan N., Farnood R. (2020). High-performance photocatalysts for the selective oxidation of alcohols to carbonyl compounds. Can. J. Chem. Eng..

[cit164] Bellotti P. (2023). *et al.*, Photocatalytic late-stage C–H functionalization. Chem. Rev..

[cit165] Zivic N. (2016). *et al.*, Photocatalysts in polymerization reactions. ChemCatChem.

[cit166] Hao T. (2025). *et al.*, Dual pyridinic nitrogen atoms as key nodes in triazine-based conjugated porous polymers toward photocatalytic hydrogen evolution. ACS Appl. Polym. Mater..

[cit167] Huang X. (2018). *et al.*, A sustainable method toward melamine-based conjugated polymer semiconductors for efficient photocatalytic hydrogen production under visible light. Green Chem..

[cit168] Wu Y. (2024). *et al.*, Facile hydrothermal synthesis of melamine-based polymers for photocatalytic hydrogen evolution. J. Saudi Chem. Soc..

[cit169] Kim S. (2026). *et al.*, Design strategies, methods, and photophysical insights in polymeric photocatalysts for solar-driven hydrogen evolution. Nano Convergence.

[cit170] Karim A. V., Krishnan S., Shriwastav A. (2022). An overview of heterogeneous photocatalysis for the degradation of organic compounds: A special emphasis on photocorrosion and reusability. J. Indian Chem. Soc..

[cit171] Xiao M. (2023). *et al.*, Addressing the stability challenge of photo (electro) catalysts towards solar water splitting. Chem. Sci..

[cit172] Fox M. A., Dulay M. T. (1993). Heterogeneous photocatalysis. Chem. Rev..

[cit173] Du F. (2024). *et al.*, Improving the stability of halide perovskites for photo-, electro-, photoelectro-chemical applications. Adv. Funct. Mater..

[cit174] Wu X. (2025). *et al.*, Toward the rational selection of safe and sustainable solvents in semiconductor photocatalysis. ChemSusChem.

[cit175] Haranaka-Funai D. (2017). *et al.*, Photocatalytic treatment of valproic acid sodium salt with TiO_2_ in different experimental devices: An economic and energetic comparison. Chem. Eng. J..

[cit176] Bolton J. R. (2001). *et al.*, Figures-of-merit for the technical development and application of advanced oxidation technologies for both electric-and solar-driven systems (IUPAC Technical Report). Pure Appl. Chem..

[cit177] Cunha I. L. C. (2026). *et al.*, Is photocatalysis ready for scale yet?. Processes.

[cit178] Thang N. H., Hien N. C. (2026). Environmental and energy applications of green-synthesized semiconductor nanomaterials: from photocatalysis to smart sensors. Ionics.

[cit179] Kumar A., Pandey G. (2017). A review on the factors affecting the photocatalytic degradation of hazardous materials. Mater. Sci. Eng. Int. J..

[cit180] Zhang P. (2016). *et al.*, Effective charge carrier utilization in photocatalytic conversions. Acc. Chem. Res..

[cit181] Lu S. (2024). *et al.*, Visible light photocatalytic performance of Co/Ag bimetallic coordination polymer catalysts designed from hard and soft acid–base theory. J. Environ. Chem. Eng..

